# Occupational burnout, flourishing and job satisfaction among HIV/AIDS healthcare workers in Western China: a network analysis

**DOI:** 10.1186/s12888-023-04959-7

**Published:** 2023-08-03

**Authors:** Siyan Jia, Bin Yu, Chuanteng Feng, Peng Jia, Peng Xu, Shujuan Yang

**Affiliations:** 1 West China School of Public Health and West China Fourth Hospital, Sichuan University, Chengdu, China; 2 Institute for Disaster Management and Reconstruction, Sichuan University, Chengdu, China; 3 School of Resource and Environmental Sciences, Wuhan University, Wuhan, China; 4 International Institute of Spatial Lifecourse Health (ISLE), Wuhan University, Wuhan, China; 5 National Center for STD/AIDS Control and Prevention, China Center for Disease Control and Prevention, Beijing, China; 6 Department of Health Management Center, Clinical Medical College & Affiliated Hospital, Chengdu University, Chengdu, China

**Keywords:** Occupational burnout, Flourishing, Job satisfaction, Network analysis, Healthcare workers

## Abstract

**Background:**

Healthcare workers suffered with high prevalence of occupational burnout, which might be related with their job satisfaction and well-being. This study aimed to provide evidence of complex interrelations among occupational burnout, flourishing, and job satisfaction, and identify key variables from the perspective of network structure among healthcare workers.

**Methods:**

A cross-sectional study was conducted between July and October 2021, and 907 (the response rate was 98.4%) HIV/AIDS healthcare workers completed their sociodemographic characteristics, occupational burnout, flourishing and job satisfaction. Network analysis was conducted to investigate the interrelations of occupational burnout, flourishing, and job satisfaction communities, and identify central variables and bridges connecting different communities with different bridge strength thresholds in the network structure. The Network Comparison Test (NCT) was conducted to examine the gender differences in networks.

**Results:**

In the network, feeling exhausted at work (strength: 1.42) and feeling frustrated at work (1.27) in occupational burnout community, and interested in daily activities (1.32) in flourishing community were central variables. Bridges in the network were job reward satisfaction (bridge strength: 0.31), satisfaction with job itself (0.25), and job environment satisfaction (0.19) in job satisfaction community, as well as interested in daily activities (0.29) and feeling respectable (0.18) in flourishing community, with bridges selected with top 20% bridge strengths. Feeling frustrated at work (0.14) in occupational burnout community and leading a purposeful and meaningful life (0.11) in flourishing community became bridges when using thresholds of top 25% and 30% bridge strengths, respectively. We also observed higher network densities in females (network density: 0.37) than that in males (0.34), and gender differences in the distribution of partial correlation coefficients (*M* = 0.27, *P* = 0.017).

**Conclusions:**

In the network structure of occupational burnout-flourishing-job satisfaction, feeling frustrated at work in occupational burnout community and interested in daily activities in flourishing community were both central variables and bridges, which may be targeted variables to intervene to alleviate the overall level of symptoms in the network and therefore prevent poor health outcomes in healthcare workers.

**Supplementary Information:**

The online version contains supplementary material available at 10.1186/s12888-023-04959-7.

## Background

Healthcare workers, who are a significant part of the health system, are well-known to experience excessive workloads [1, 2]. Overloaded work, high work-related demand and unsupportive environments can induce healthcare workers’ occupational burnout [3]. Rotenstein and colleagues estimated a pooled prevalence of 21.3% on occupational burnout in practicing physicians [4]. In Chinese healthcare workers, related studies also showed a high prevalence of occupational burnout ranging from 75.5% to 80.0% [5–7], and the prevalence in HIV/AIDS healthcare workers has reached 76.9% [8]. The ratio between healthcare workers and patients was imbalanced because of a serious shortage of HIV/AIDS healthcare workers in China [8]. In addition, HIV/AIDS healthcare workers usually face occupational exposure risks for HIV infection, which may aggravate their occupational burnout [9]. Occupational burnout is one of the critical reasons for poor quality of medical services, medical accidents, and low efficacy of job performance [10–15]. It is urgent to understand occupational burnout and its influencing factors especially among HIV/AIDS healthcare workers.

Occupational burnout may be caused by negative psychological factors, such as anxiety, depression, and poor aptitude for interpersonal relationships [16, 17]. In contrast, positive psychological factors are beneficial in reducing occupational burnout [18]. Flourishing, a state in which all aspects of one’s experience are well, could reflect overall well-being comprehensively and has more advantages in explaining complex relations and connotations compared with other tools [19, 20]. Employees with flourishing would exhibit positive outcomes, such as higher job satisfaction [21–25]. To date, evidence on flourishing and the association between flourishing and occupation burnout in HIV/AIDS healthcare workers is scarce.

Job satisfaction, defined as people's feelings about their jobs [26], is another factor that would greatly increase enthusiasm and reduce the absenteeism of workers, even in those with high occupational burnout [27–32]. Job satisfaction was related to positive psychological factors [33, 34]. As described in the spillover model, higher job satisfaction leads to higher well-being [35]. However, current studies estimated the relationships between these positive and negative psychological factors (i.e., occupational burnout, flourishing, and job satisfaction) based on the sum-scores of scales rather than individual items, and the subtle interrelationships among the dimensions of job satisfaction, flourishing and occupational burnout among HIV/AIDS healthcare workers remains to be elucidated clearly.

Network analysis has increasingly been used in psychological research to quantify the relationships among multi-dimensional psychological factors and identify the most interconnected dimensions at a time. Meanwhile, it is plausible that a group of nodes (variables) belong to a community (i.e., the same rating scale) where they are closely related, and network models can help identify central variables in these closely related variables [36]. Moreover, network models describe an interacting web of symptoms and could help to understand the transdiagnostic symptom relationships that underlie the well-established association of occupational burnout, flourishing and job satisfaction, and can be a useful method to examine the bridges that are represented by one or more key variables bridging two or more communities [37]. Deactivating bridges may constitute an effective strategy to block connections between items efficiently. For example, a previous study identified bridge symptoms (i.e., “irritability”, “feeling afraid” and “sad mood”) that could be targeted in specific treatment and preventive measures for comorbid depressive and anxiety symptoms [38]. However, limited research, to our knowledge, used this method in occupational populations who have a high probability of occupational burnout and other negative emotions.

In light of the above gaps, we used network analysis to (1) estimate the associations of occupational burnout, flourishing, and job satisfaction in a constructed network; (2) identify central variables in network structure of occupational burnout-flourishing-job satisfaction and (3) seek bridges linking occupational burnout, flourishing and job satisfaction. Previous studies found that there were differences between males and females in the prevalence of occupational burnout and mental health, and attitudes toward job satisfaction [39, 40]. We therefore test the differences in the network structure of occupational burnout-flourishing-job satisfaction in males and females. The findings of this study would help obtain a straightforward view of variables’ interactions to clarify critical issues and guide more effective interventions to improve the health and professional happiness of healthcare workers in China. More broadly, our findings would help policy-making for improving the psychological health of other healthcare workers.

## Material and methods

### Study design and participants

This study is an observational and cross-sectional design in the Sichuan province of China, where is one of the most HIV-affected province in China [41, 42]. In such a scenario, HIV/AIDS healthcare workers in Sichuan province may undertake overloaded work and high work-related demands. The current study used a stratified cluster sampling method to obtain a sample. First, we randomly selected three cities based on the prevalence of HIV as our study sites. Second, all related healthcare workers in selected cities were recruited for investigation (e.g., from the Centers for Disease Control and Prevention (CDC) and Designated Hospitals for HIV treatment) (Figure S1). Finally, 922 HIV/AIDS healthcare workers were enrolled to complete the electronic survey. Inclusion criteria of the study included at least 18 years old at the time of entry to this study, medical and management personnel involved in HIV/AIDS prevention and control work. Exclusion criteria included local HIV/AIDS health workers who were illiterate, and those who refused to participate in this survey. All the participants gave informed assent forms before data collection; it has been stated on the cover page that the participants cannot proceed with the online survey without consent. Ethics approval was obtained from the Ethics Committee West China School of Public Health and West China Fourth Hospital, Sichuan University (Gwll2021059).

The sample size was calculated based on $n=\frac{{\mu }_{\alpha /2}^{2}\pi (1-\pi )}{{\delta }^{2}}$. $\pi =76.9\%$ (76.9% of HIV/AIDS healthcare workers met the criteria for burnout in a province of China) [8], $\delta =0.05, \alpha =0.05, {\mu }_{\alpha /2}=1.96,$ and the sample size was calculated to be 273. However, due to the large sampling error of the cluster, the sample size was usually 1.5 times of the sample size of the pure random sample, so the sample size of participants should be at least 409.

### Data collection

Data for the analysis was collected between July 2021and October 2021 through an online questionnaire designed by an expert panel. The panel consisted of two epidemiologists, one health psychologist, and two HIV/AIDS healthcare workers. A pretest was conducted in 10 HIV/AIDS healthcare workers to test the questionnaire's comprehension and the feasibility of investigation process. Their feedback was used to revise and finalize the questionnaire. Besides, the online questionnaire with attached instructions for completion was released by the HIV/AIDS management department in the Health Commission of the three cities through WeChat and other social media platforms and forwarded to all the HIV/AIDS healthcare workers. The questionnaire took about 20 min to complete on average.

### Measurement

#### Occupational burnout scale

The widely used scale to measure occupational burnout is the version of the Maslach Burnout Inventory-General Survey (MBI-GS) by Maslach and Jackson [43], which has good reliability and validity in China [44]. Our study used a self-designed scale developed based on the MBI-GS to better adapt the local culture of HIV/AIDS healthcare workers, and to ensure the high-quality results, that is, within the endurance limits to be interviewed. The scale used 11 items to assess the occurrence of occupational burnout, including emotional exhaustion (3 items), work fatigue (1 item), emotional numbness (3 items), work frustration (2 items), and work pressure (2 items), and it was a five-point scale ranging from strongly disagree (1-point) to strongly agree (5-point). A sum score ranging from 11 to 55 was calculated, and higher scores indicated more severe occupational burnout (Table S1). The items in occupational burnout scale specifically described the individual's experiences and feelings about burnout. By using these items as analyzed variables, we could capture individual responses to different aspects of burnout, and further understand the network structure and characteristics of burnout. The occupational burnout scale demonstrated high internal consistency, with Cronbach’s alpha of 0.895 in this study. The KMO coefficient was 0.878, and Bartlett's sphericity test was significant (*P* < 0.05), indicating good construct validity for the scale.

#### Flourishing scale

The flourishing scale (FS) was used to assess the flourishing of HIV/AIDS healthcare workers. The flourishing scale consists of 8 items describing essential aspects of human functioning and human needs, including the meaning of life, interpersonal relationship, life and work engagement, helpful to others, competence, self-esteem, optimism and respect from others [45]. Each item was answered on a 7-item scale ranging from strong disagreement (1-point) to strong agreement (7-point). The total score of all items ranged from 8 (strong disagreement) to 56 (strong agreement). A higher total score indicated a greater sense of thriving, which showed a person had a positive attitude toward life and owed many psychological resources and advantages (Table S2). The Chinese version of flourishing scale has good validity and reliability [46, 47]. The flourishing scale had high internal consistency with Cronbach’s alpha of 0.932 in this study. The KMO coefficient was 0.919, and Bartlett's sphericity test was significant (*P* < 0.05), indicating good construct validity for the scale.

#### Job satisfaction scale

Job satisfaction was measured by a self-developed scale and was revised and finalized after the pilot study. Three dimensions of job satisfaction, including satisfaction with job itself (10 items), job environment satisfaction (6 items) and job reward satisfaction (8 items), were recorded. All items had five degrees ranging from disagree (1-point) to agree (5-point). The total score of each dimension was calculated, and a higher score indicated higher job satisfaction in the corresponding dimension (Table S3). The job satisfaction scale consisted of 24 items of three dimensions. Since more variables may increase the difficulty of interpretation and lead to over-fitting and very unstable estimates in the network analysis [48], we therefore used dimensions to estimate the individual's job satisfaction of different aspects, increasing the visualization and interpretability of the results. The Cronbach’s alpha coefficients for the three dimensions of job satisfaction were 0.853, 0.834 and 0.808 in this study, respectively. The KMO coefficient for the three dimensions were 0.858, 0.841 and 0.812, respectively, and Bartlett's sphericity test was significant for each dimension of the scale (*P* < 0.05).

#### Socio-demographic and work-related characteristics

The collected socio-demographic characteristics included age, sex, marital status, educational level, personal monthly income and living situation. Besides, work-related factor included types of institutions. All relevant descriptions and explanations were detailed in Table S4.

### Statistical analysis

#### Descriptive statistics

Data were summarized by descriptive statistics, with frequency and percentage for categorical variables, and median and interquartile range (*IQR*) for continuous variables (Table S5). R version 4.0.3 for Windows was used for data management and all statistical analyses, with *P* value < 0.05 indicating statistical significance.

#### Scale-construction process (construct validity and reliability)

For the self-designed scales, psychometric analyses were performed in several steps: the first step consisted of removing items with a rate of missing values (missing value and “I don’t know” choice) > 20%, or a floor or ceiling effect > 50% [49], and no item was removed. Second, Kaiser-Meyer-Olkin (KMO) coefficients and Cronbach’s alpha coefficients were computed for evaluating the validity and internal consistency of scales, respectively. Third, an exploratory principal component analysis (PCA) using a varimax rotation for the remaining items of scales such as the job satisfaction scale was used in the study [49]. The number of dimensions was determined using a scree plot, and factor loading more than 0.60 was attributed to as a dimension [50].

#### Network estimation

We assessed the associations among occupational burnout, flourishing and job satisfaction based on the network of Graphical Gaussian Model (GGM). Partial correlation analysis was conducted to indicate the association of each pairwise variable and form networks; nodes in the network represented variables, and edges represented partial correlation coefficients between two variables. Stronger correlations were shown in thicker and more saturated edges. Positive and negative correlations were shown in green and red, respectively. In this network estimation, an Extended Bayesian Information Criterion (EBIC) model with the least absolute shrinkage and selection operator (LASSO) were used to get a sparse and intelligible network [51, 52]. We also estimated the predictability, the upper bound of variance (measured in R^2^) of a given variable explained by all the other variables in the network [53].

#### Centrality and bridge symptoms

For the constructed networks, we calculated strength centrality (i.e., the sum of the absolute value of all partial correlation coefficients for a given variable) to identify the most central variables, using the *centralityplot* function in the *qgraph* package in R [54]. In addition, we conducted a stability and reliability analysis of our results using a commonly applied bootstrapping procedure in R with *bootnet* package, which showed whether the networks remained stable when dropping 75% of the sample, and whether the results of bootstrap 95% confidence interval (*CI*) or edges were narrow, indicating the trustworthy of edges [55].

We used the term community to designate a group of items or dimensions, which were supposed to be related according to scale classification, independent of the actual network structure [36]. We also estimated variables that acted as bridges connecting communities, which allowed us to identify which items were the most interconnected across occupational burnout, flourishing and job satisfaction communities. The bridges were estimated with different scoring variable thresholds (i.e., variables that are most strongly connected to all the variables of different communities) using the *bridge* function in the *networktools* package in R [56].

#### Network comparison test

Network Comparison Test (NCT) is a permutation‐based hypothesis test for invariance of network structure (i.e., how the connections between variables within a network differ across samples) and global strength of connections (i.e., how the density of the network differs across samples – the sum of all edge strengths). To examine sex differences in the structure of networks, we ran the NCT using the *Network Comparison Test* package in R [57]. We compared the distribution of partial correlation coefficients in each network to characterize the network structure. Then, we compared differences in strength for each edge of networks between males and females, after controlling for multiple tests using a Holm-Bonferroni correction. Additionally, the network densities (the actual number of connections between variables in the network / the theoretical number of connections between variables in the network [58]) between males and females were also compared.

## Results

### Sociodemographic and work characteristics

A total of 907 (the response rate was 98.4%) HIV/AIDS healthcare workers were included in the final analysis, with a mean age of 38.0 ± 9.4 years. Among them, 69.1% (*n* = 627) were females, 49.5% (*n* = 449) graduated from junior colleges and 27.9% (*n* = 253) had a personal income below 3,000 Renminbi (RMB) per month. Further details were provided in Table 1.

**Table 1:** Baseline characteristics of the participants

Variables	N	%
**Age (years)**		
18–29	224	24.7
30–39	300	33.1
40–49	253	27.9
≥ 50	130	14.3
**Sex**		
Female	627	69.1
Male	280	30.9
**Marital status**		
Unmarried	118	13.0
Married/Living together	706	77.8
Divorce/Widowed/Living separately	83	9.2
**Educational level**		
High school or below	123	13.6
Junior college	449	49.5
Undergraduate or above	335	36.9
**Personal monthly income (RMB)**		
< 3000	253	27.9
3000–4000	304	33.5
4000–5000	176	19.4
≥ 5000	174	19.2
**Living situation**		
Alone	74	8.2
With friends or others	41	4.5
With parents	792	87.3
**Types of institution**		
CDC	91	10.0
Designated Hospital for treatment	105	11.6
County Governments	95	10.4
Community Health Service Centers	591	65.2
Others^a^	25	2.8

*Abbreviations*: *CDC* Centers for Disease Control and Prevention; *RMB* renminbi

^a^Others is a self-selected designation that indicates the institution is not listed

### Network structure and centrality measures

**Fig. 1:**
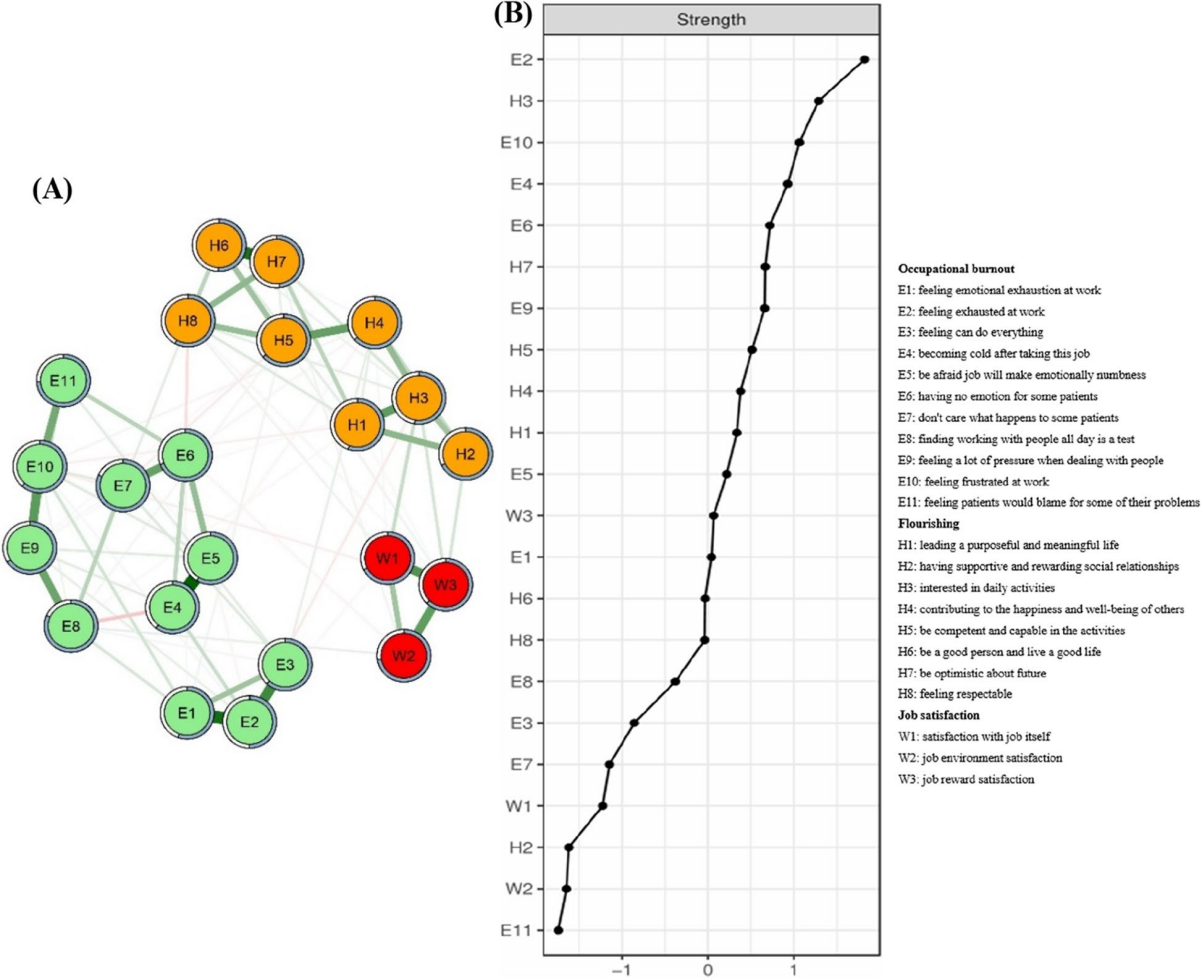
Networks and strength centrality displaying the relationship between occupational burnout, flourishing and job satisfaction. **A** The regularized network between occupational burnout, flourishing and job satisfaction. **B** Standardized variable strength centrality for the network (z-scores). Variables are represented by nodes, numbers represent item numbers in the scale, partial correlations between the variables are represented by edges. The color of the edge indicates the direction of the correlation (red = negative, green = positive). The magnitude of the edge depicts the magnitude of the correlation, with thicker and saturated edges showing stronger correlations. Positive and negative correlations were shown in green and red, respectively. Variables identified as job satisfaction are colored in red circles; Variables identified as flourishing are colored in orange circles; Variables identified as occupational burnout are colored in green circles. The area in the rings around the variables indicates predictability (the upper bound of the variance of a given variable explained by the remaining variables in the network)

**Fig. 2:**
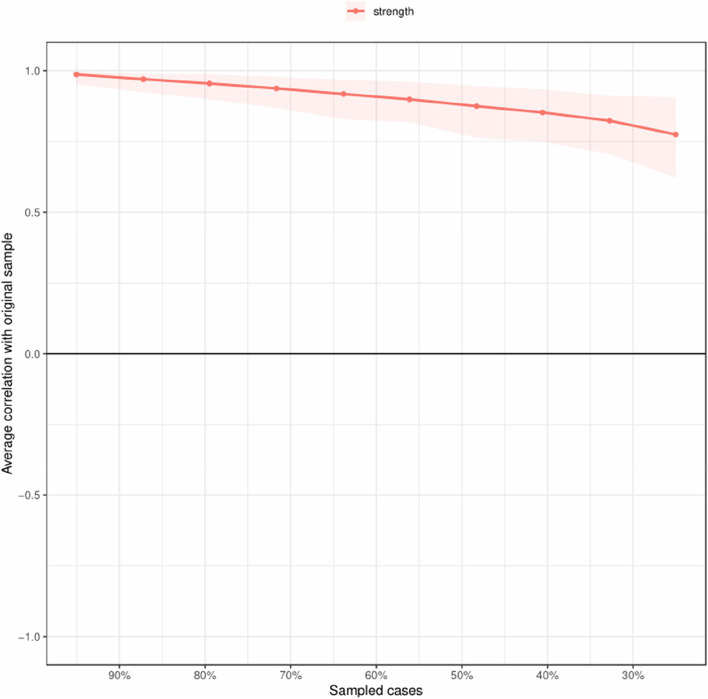
Stability of centrality indices by case dropping bootstrap for the network shown in Fig. 1 (The bootstrap was repeated 1000 times). The x-axis represents the percentage of cases of original sample used at each step. The y-axis represents the average of correlations between the centrality indices from the original network and the centrality indices from the networks that were re-estimated after dropping increasing percentages of cases. Each line indicates the correlations of strength, while areas indicate 95%*CI*

The network structure showed a high degree of interrelations between variables from occupational burnout, flourishing and job satisfaction communities, and the variables in the same communities tended to cluster together. Most of the associations between variables of different communities were negative (Fig. 1). The predictabilities of variables were shown as ring shaped pie charts, and variables with the highest centrality were E2 (feeling exhausted at work in occupational burnout community; strength: 1.42), H3 (interested in daily activities in flourishing community; strength: 1.32) and E10 (feeling frustrated at work in occupational burnout community; strength: 1.27). We also found the stability of the network remained stable (i.e., case-dropping coefficient = 0.75) even dropping large proportions of the sample (Fig. 2). The numerical interactions were shown in Table S6 using weighted adjacency matrix that represented the strength of associations between variables. The results of the reliability and stability analyses were presented in Figures S2, S3 and S4.

### Bridges and bridge centrality measures

**Fig. 3:**
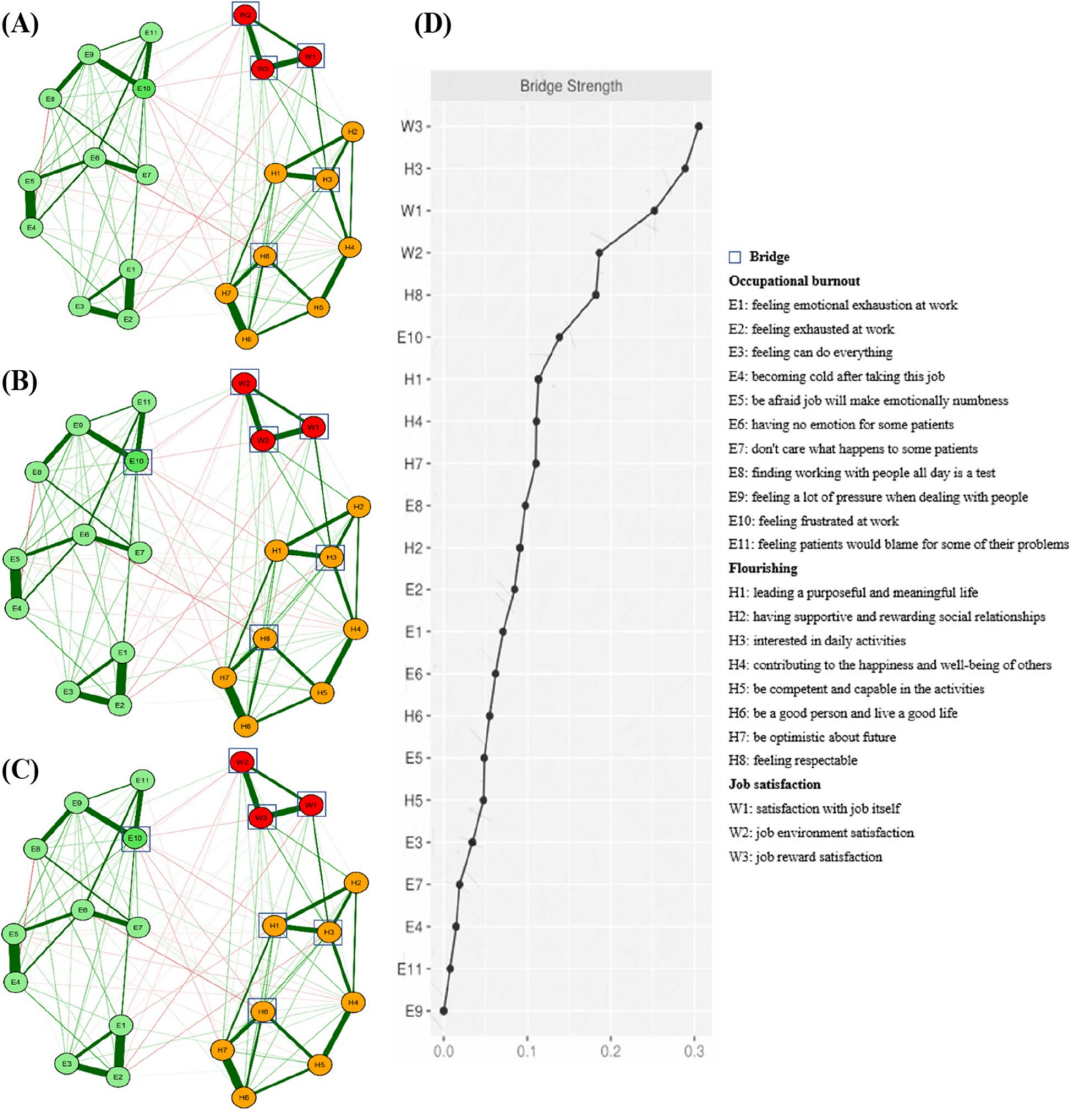
Networks and bridge strength centrality displaying the relationship between occupational burnout, flourishing and job satisfaction. **A** The top 20% scoring variables on a given statistic and selected as predicted bridges. **B** The top 25% scoring variables on a given statistic and selected as predicted bridges. **C** The top 30% scoring variables on a given statistic and selected as predicted bridges. **D** Bridge centrality estimates for each variable in the network, ordered by highest value. Variables are represented by nodes, numbers represent item numbers in the scale, partial correlations between the variables are represent by edges. The color of the edge indicates the direction of the correlation (red = negative, green = positive). The magnitude of the edge depicts the magnitude of the correlation, with thicker and saturated edges showing stronger correlations. Positive and negative correlations were shown in green and red, respectively. Variables identified as job satisfaction are colored in red circle; Variables identified as flourishing are colored in orange circle; Variables identified as occupational burnout are colored in green circle; Variables identified as bridges are drew with rectangles

Figure 3 showed the bridges and bridge centrality indices. The top 20% scoring variables on bridge strength were W1 (satisfaction with job itself; bridge strength: 0.25), W2 (job environment satisfaction; bridge strength: 0.19) and W3 (job reward satisfaction; bridge strength: 0.31) in job satisfaction community; H3 (interested in daily activities; bridge strength: 0.29) and H8 (feeling respectable; bridge strength: 0.18) in flourishing community (Fig. 3A). When identifying bridges with the top 25% bridge strengths, we observed that E10 (feeling frustrated at work; bridge strength: 0.14) in the occupational burnout community was additionally added as a bridge linking other communities (Fig. 3B). Furthermore, H1 (leading a purposeful and meaningful life of flourishing community; bridge strength: 0.11) was added as a bridge when using the top 30% bridge strengths (Fig. 3C). Bridge strength was reported in Fig. 3D.

### Network comparisons between males and females

**Fig. 4:**
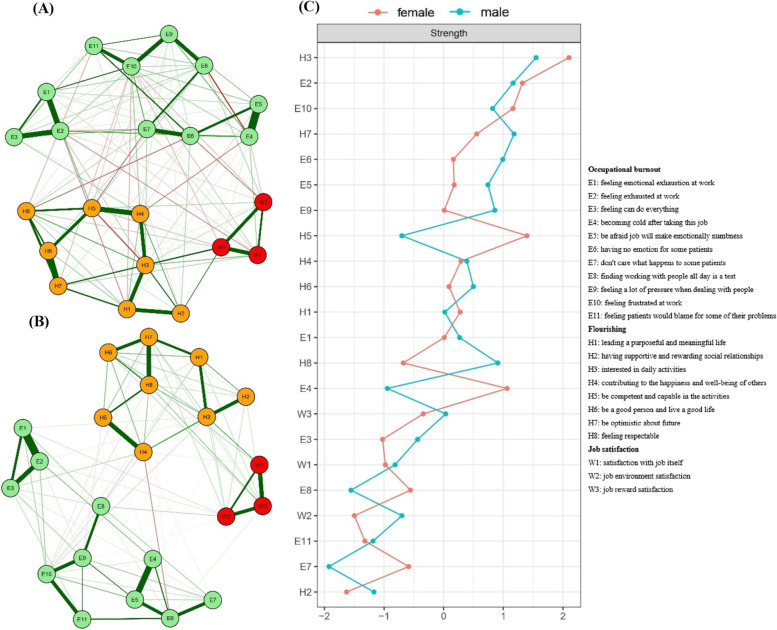
Networks and strength centrality displaying the relationship between occupational burnout, flourishing and job satisfaction in males and females. **A** Graphical LASSO model of variables with occupational burnout, flourishing and job satisfaction in females (*n* = 627). **B** Graphical LASSO model of variables with occupational burnout, flourishing and job satisfaction in males (*n* = 280). **C** Comparison of network centrality indices between males and females. Variables are represented by nodes, numbers represent item numbers in the scale, partial correlations between the variables are represented by edges. The color of the edge indicates the direction of the correlation (red = negative, green = positive). The magnitude of the edge depicts the magnitude of the correlation, with thicker and saturated edges showing stronger correlations. Positive and negative correlations were shown in green and red, respectively. Variables identified as job satisfaction are colored in red circles; Variables identified as flourishing are colored in orange circles; Variables identified as occupational burnout are colored in green circles

The network structures and network centrality indices of males (*n* = 607) and females (*n* = 280) were showed in Fig. 4, and the network structures were both stable (Figures S5, S6, S7 and S8). The number of edges in female network were 86, and in male network were 79. The network density of males and females were 0.37 and 0.34, respectively. The results of the NCT indicated significant sex differences in the distribution of partial correlation coefficients (*M* = 0.27, *P* = 0.017), and no significant gender differences were observed in network global strength (males: 9.91 vs. females: 10.63; global strength difference = 0.72, *P* = 0.071) (Figure S9).

**Fig. 5:**
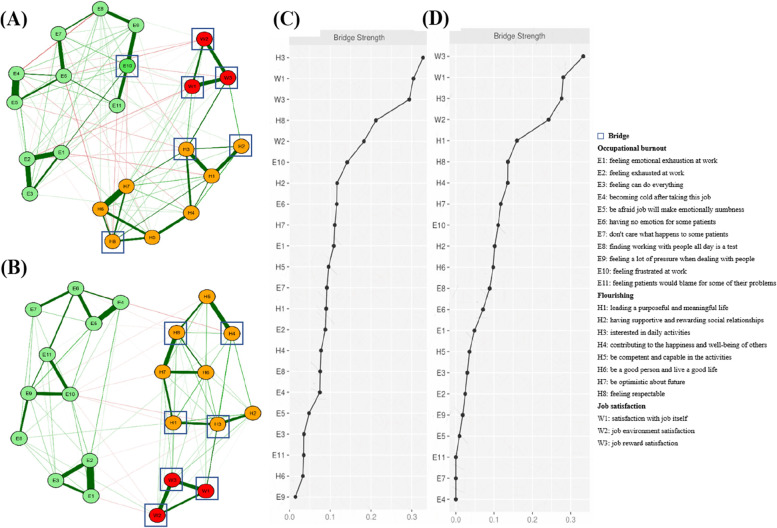
Networks and bridge strength centrality displaying the relationship between occupational burnout, flourishing and job satisfaction in males and females. **A** Graphical LASSO model of variables with occupational burnout, flourishing and job satisfaction in females of the sample (*n* = 687). **B** Graphical LASSO model of variables with job satisfaction, flourishing and occupational burnout in males of the sample (*n* = 280). **C** Bridge centrality estimates for each variable in the network of females, ordered by highest value. **D** Bridge centrality estimates for each variable in the network of males, ordered by highest value. Variables are represented by nodes, numbers represent item numbers in the scale, and partial correlations between the variables are represented by edges. The color of the edge indicates the direction of the correlation (red = negative, green = positive). The magnitude of the edge depicts the magnitude of the correlation, with thicker and saturated edges showing stronger correlations. Positive and negative correlations were shown in green and red, respectively. Variables identified as job satisfaction are colored in red circles; Variables identified as flourishing are colored in orange circles; Variables identified as occupational burnout are colored in green circles; Variables identified as bridges are drawn with rectangles. The top 30% scoring variables on a given statistic and selected as predicted bridges in the males and females

We also calculated bridge centrality in males and females’ networks, separately (Fig. 5). No bridge was found with the top 20% and 25% bridge strengths, but seven bridges were found in both males and females’ networks with the top 30% bridge strength. However, there was a significant sex difference. For example, E10 (feeling frustrated at work of occupational burnout community; bridge strength: 0.14) was a bridge in the network of females, but no bridge was found in occupational burnout community in the network of males. Besides, H2 (having supportive and rewarding social relationships; bridge strength: 0.12) was a bridge in flourishing community in females’ network, however, H1 (leading a purposeful and meaningful life; bridge strength: 0.09) and H4 (contributing to the happiness and well-being of others; bridge strength: 0.08) were bridges in males’ network.

## Discussion

This study estimated the network structure of occupational burnout, flourishing and job satisfaction, and firstly attempted to assess their interactions at the item level in HIV/AIDS healthcare workers. Our results indicated that variables feeling exhausted at work and feeling frustrated at work in occupational burnout community, and interested in daily activities in flourishing community were the most central variables. Job reward satisfaction, satisfaction with job itself and job environment satisfaction in job satisfaction community, as well as interested in daily activities and feeling respectable in flourishing community were bridges when identifying bridges with the top 20% bridge strengths. Feeling frustrated at work in the occupational burnout community and leading a purposeful and meaningful life in the flourishing community became bridges connecting other communities with the top 25% and 30% bridge strengths, respectively. Gender was associated with different distributions of partial correlation coefficients between their networks, and network densities in females were higher than that in males.

Central variables may give insights into the connectedness or importance of items within a network and may play a major role in causing the onset of and/or maintaining a syndrome [59]. Bridges mediate the transition among different syndromes and may increase the risk of contagion to other disorders [60]. In this study, feeling exhausted at work was the most central variable with high centrality in the network, followed by interested in daily activities and feeling frustrated at work. Meanwhile, we found that interested in daily activities and feeling frustrated at work were also bridges connecting other communities. Thus, there may have some overlaps between central variables and bridges, and these overlaps deserve special attention. A potential explanation is that interested in daily activities and feeling frustrated at work are two key mental statuses of coexistence that may affect other mental statuses. For HIV/AIDS healthcare workers in charge of disease prevention and control and patients' treatment, they are overburdened with heavy work demands, and the mismatch between effort and income may make them frustrated [61]. Besides, previous studies showed that some healthcare workers in special departments experienced more psychological pressure, that is, much of the pressure mainly comes from daily activities [62, 63]. As Sichuan Province is one of the provinces with high HIV epidemic in China, the more attention AIDS prevention and treatment receives, the greater the responsibility HIV/AIDS healthcare workers assume. In addition, if the occupational population is interested in their daily work, they may have high job satisfaction and be less prone to occupational burnout since they would think the work being an emotional reward, and may be more motivated to complete [64]. These central variables and bridges not only played an important role in understanding the structure of the network model, but also can serve as explicit intervention points to relieve all symptoms. It should be noted that bridges changed in different percentile cut-offs of bridge strength (80%, 75%, and 70%), which were different from Jones's study that only focused on one cut-off [36]. The number of bridges would increase when the percentile cut-off point decreased, and therefore we identified at least one bridge from each community, which may guide us to carry out targeted interventions in the bridges corresponding to each community.

In this study, the items belonging to the same communities tended to cluster together, and the relationships between occupational burnout, flourishing and job satisfaction were more subtle compared to previous studies' findings that only considered the total score or subscale total scores of the rating scales [21, 65, 66]. Meanwhile, our study revealed that there were intrinsic interactions among variables of different communities. For example, leading a purposeful and meaningful life in flourishing community and feeling frustrated at work in occupational burnout community were related, which was consistent with a previous study [67]. That is, personal value and a sense of self-fulfilled could help individuals find meaning and value in their work by increasing their satisfaction and sense of accomplishment at work, thereby reducing occupational burnout. Therefore, network analysis offers an additional tool to capture connecting patterns of occupational burnout, flourishing and job satisfaction, and intrinsic connections among variables of different communities suggesting that interventions targeting crucial variables in each community may be beneficial to the whole network.

Furthermore, our analysis documented both similarities and differences in the network structure of occupational burnout-flourishing-job satisfaction in males and females. The central variables were almost similar in both males and females. However, the bridges had a large difference in occupational burnout and flourishing community, which may be caused by the career expectations and attitudes towards problems between males and females [68]. We also examined sex differences in the structure of networks and found network densities between males and females were different. Network density describes the portion of the potential connections in a network that are actual connections and refers to the mean strength of the connections between variables in networks [58]. In the study, we found higher network densities in females than that in males, which showed that symptoms may transmit more quickly in females. The results were similar to previous studies which have found that males and females responded to conditions in different ways and females tend to develop internal symptoms [39, 69]. The differences may be derived from particular psychological characteristics, unique perspectives and experiences on work-related aspects in females which were different from males.

This study has several strengths. First, the network model was designed to consider individual symptoms as variables in the study. In addition, we compared bridges under different thresholds, which can help to find more important bridges in different communities and help design subtle interventions. Finally, NCT analysis explicitly tested gender as a sociodemographic influence on the network structure of occupational burnout-flourishing-job satisfaction of health workers, and indicated the heterogeneity in central and bridges for targeted intervention in two networks.

Several limitations should also be mentioned. First, the cross-sectional data is limited to infer causal relations. To fully conceptualize how the variables interact with one another, future experimental and longitudinal studies are needed. However, related research has shown that network structures among cross-sectional and longitudinal studies did not differ [70]. Second, as the study relied on the self-report online questionnaire, the results may not rule out other possible factors, social desirability or recall biases, etc. There may be also understanding deviations of different respondents and the reliability and accuracy of the survey may be affected to some extent. Third, we only examined the difference in network structure between gender. However, other sociodemographic factors are also needed to examine in future studies. Finally, generalizations should be taken with caution since the sample comprised only a small proportion of healthcare workers in China. Therefore, researches on geographical and cultural diversity are warranted in the future.

## Conclusions

Through network analysis, we estimated the complex association between occupational burnout, flourishing and job satisfaction among Chinese healthcare workers. Feeling frustrated at work of occupational burnout community and interested in daily activities of flourishing community were both the central variables and bridges. Specific strategies and measures targeting to these variables should be given such as improving levels of flourishing and job satisfaction and reducing occupational burnout. Additionally, government and healthcare agencies should address occupational burnout and improve flourishing, especially take initiatives to improve job satisfaction with effective interventions in healthcare workers.

### Supplementary Information

**Additional file 1: Table S1.** Description of occupational burnout scale. **Table S2.** Description of flourishing scale. **Table S3.** Description of job satisfaction scale. **Table S4.** Description of the key variables used in this study. **Table S5.** Baseline characteristics and medians (IQR) for occupational burnout, job satisfaction and flourishing of the participants. **Table S6.** Weighted adjacency matrix. **Figure S1.** Flowchart of the study sample. **Figure S2.** Bootstrap 95% confidence intervals of the partial correlation coefficients. **Figure S3.** Estimation of edge weight differences by bootstrapped difference test. **Figure S4.** Bootstrap partial correlation coefficients difference test of the node’s strength. **Figure S5.** Stability of centrality indices by case dropping bootstrap in males and females. **Figure S6.** Bootstrap 95% confidence intervals of partial correlation coefficients of the network in males and females. **Figure S7.** Estimation of edge weight difference in males and females. **Figure S8.** Bootstrap partial correlation coefficients difference test of node’s strength in males and females. **Figure S9.** Comparison of network properties in males and females

## Data Availability

The datasets used and/or analysed during the current study are available from the corresponding author on reasonable request.

## References

[1] Anyangwe SC, Mtonga C (2007). Inequities in the global health workforce: the greatest impediment to health in Sub-saharan Africa. Int J Environ Res Public Health.

[2] West CP, Dyrbye LN, Shanafelt TD (2018). Physician burnout: contributors, consequences and solutions. J Intern Med.

[3] Sikka R, Morath JM, Leape L (2015). The quadruple aim: care, health, cost and meaning in work. BMJ Qual Saf.

[4] Rotenstein LS, Torre M, Ramos MA, Rosales RC, Guille C, Sen S (2018). Prevalence of burnout among physicians: a systematic review. JAMA.

[5] Chen J, Cao X, Wu Z (2015). Research progress in job burnout among hiv-related health care workers. Zhonghua Liu Xing Bing Xue Za Zhi.

[6] Chunmai D, Congbin Z, Bin W, Ruimin Z (2013). Study on job burnout among employees in methadone maintenance treatment clinic. Chinese J Drug Abuse Prev Treat.

[7] Qingling C, Huiqin L, Sha Z, Siyun F, Jincheng L (2013). Study on job burnout among medical staff in caring for HIV/AIDS. Chin J AIDS HIV.

[8] Qiao Z, Chen L, Chen M, Guan X, Wang L, Jiao Y (2016). Prevalence and factors associated with occupational burnout among HIV/AIDS healthcare workers in China: a cross-sectional study. BMC Public Health.

[9] Sanchez TH, Kelley CF, Rosenberg E, Luisi N, O'Hara B, Lambert R (2014). Lack of awareness of human immunodeficiency virus (HIV) infection: problems and solutions with self-reported HIV serostatus of men who have sex with men. Open Forum Infect Dis.

[10] Dewa CS, Loong D, Bonato S, Trojanowski L (2017). The relationship between physician burnout and quality of healthcare in terms of safety and acceptability: a systematic review. BMJ Open.

[11] Panagioti M, Geraghty K, Johnson J, Zhou A, Panagopoulou E, Chew-Graham C (2018). Association between physician burnout and patient safety, professionalism, and patient satisfaction: a systematic review and meta-analysis. Jama Intern Med.

[12] Perez-Francisco DH, Duarte-Climents G, Del RJ, Gomez-Salgado J, Romero-Martin M, Sanchez-Gomez MB (2020). Influence of workload on primary care nurses ‘health and burnout, patients’ safety, and quality of care: integrative review. Healthcare.

[13] Salyers MP, Bonfils KA, Luther L, Firmin RL, White DA, Adams EL (2017). The relationship between professional burnout and quality and safety in healthcare: a meta-analysis. J Gen Intern Med.

[14] Seo HS, Kim H, Hwang SM, Hong SH, Lee IY (2016). Predictors of job satisfaction and burnout among tuberculosis management nurses and physicians. Epidemiol Health.

[15] Suñer-Soler R, Grau-Martín A, Flichtentrei D, Prats M, Braga F, Font-Mayolas S (2014). The consequences of burnout syndrome among healthcare professionals in Spain and Spanish speaking Latin American countries. Burn Res.

[16] Bellani ML, Furlani F, Gnecchi M, Pezzotta P, Trotti EM, Bellotti GG (1996). Burnout and related factors among HIV.AIDS health care workers. Aids Care.

[17] Hayter M (1999). Burnout and aids care-related factors in HIV community clinical nurse specialists in the north of England. J Adv Nurs.

[18] Mascaro JS, Wallace A, Hyman B, Haack C, Hill CC, Moore MA (2022). Flourishing in healthcare trainees: psychological well-being and the conserved transcriptional response to adversity. Int J Environ Res Public Health.

[19] Keyes CL (2007). Promoting and protecting mental health as flourishing: a complementary strategy for improving national mental health. Am Psychol.

[20] Rump S (2015). The flourishing scale in comparison with other well-being scales: the examination and validation of a new measure.

[21] Vetter MH, Vetter MK, Fowler J (2018). Resilience, hope and flourishing are inversely associated with burnout among members of the society for gynecologic oncology. Gynecol Oncol Rep.

[22] Freire C, Ferradas M, Garcia-Bertoa A, Nunez JC, Rodriguez S, Pineiro I (2020). Psychological capital and burnout in teachers: the mediating role of flourishing. Int J Environ Res Public Health.

[23] Bowling NA, Eschleman KJ, Wang Q (2010). A meta-analytic examination of the relationship between job satisfaction and subjective well-being. J Occup Organ Psychol.

[24] Naehrig D, Schokman A, Hughes JK, Epstein R, Hickie IB, Glozier N (2021). Effect of interventions for the well-being, satisfaction and flourishing of general practitioners-a systematic review. BMJ Open.

[25] Diedericks E, Rothmann S (2014). Flourishing of information technology professionals: effects on individual and organisational outcomes. S Afr J Bus Manag.

[26] Spector PE (1997). Job satisfaction: application, assessment, causes, and consequences.

[27] Harris RV, Ashcroft A, Burnside G, Dancer JM, Smith D, Grieveson B (2008). Facets of job satisfaction of dental practitioners working in different organisational settings in England. Br Dent J.

[28] Kim MH, Mazenga AC, Simon K, Yu X, Ahmed S, Nyasulu P (2018). Burnout and self-reported suboptimal patient care amongst health care workers providing HIV care in Malawi. Plos One.

[29] Omolase CO, Seidu MA, Omolase BO, Agborubere DE (2010). Job satisfaction amongst nigerian ophthalmologists: an exploratory study. Libyan J Med..

[30] Wang H, Jin Y, Wang D, Zhao S, Sang X, Yuan B (2020). Job satisfaction, burnout, and turnover intention among primary care providers in rural China: results from structural equation modeling. BMC Fam Pract.

[31] Sancho FM, Ruiz CN (2010). Risk of suicide amongst dentists: myth or reality?. Int Dent J.

[32] Scanlan JN, Still M, Radican J, Henkel D, Heffernan T, Farrugia P (2020). Workplace experiences of mental health consumer peer workers in new south Wales, Australia: a survey study exploring job satisfaction, burnout and turnover intention. BMC Psychiatry.

[33] Xiao L. The impact of internet employees’ psychological capital on job burnout: mediation effect of job satisfaction and moderating effect of support and uncontrolled management. Hangzhou, Zhejiang: Zhejiang University press; 2019.

[34] Yue Z, Qin Y, Li Y, Wang J, Nicholas S, Maitland E (2022). Empathy and burnout in medical staff: mediating role of job satisfaction and job commitment. BMC Public Health.

[35] Dolan P, Peasgood T, White M (2008). Do we really know what makes us happy? A review of the economic literature on the factors associated with subjective well-being. J Econ Psychol.

[36] Jones PJ, Ma R, McNally RJ (2021). Bridge centrality: a network approach to understanding comorbidity. Multivariate Behav Res.

[37] Triolo F, BelvederiMurri M, Calderón-Larrañaga A, Vetrano DL, Sjöberg L, Fratiglioni L (2021). Bridging late-life depression and chronic somatic diseases: a network analysis. Transl Psychiatry.

[38] Jin Y, Sha S, Tian T, Wang Q, Liang S, Wang Z (2022). Network analysis of comorbid depression and anxiety and their associations with quality of life among clinicians in public hospitals during the late stage of the covid-19 pandemic in China. J Affect Disord.

[39] Hill TD, Needham BL (2013). Rethinking gender and mental health: a critical analysis of three propositions. Soc Sci Med.

[40] Haller G, Delhumeau C, Mamie C, Zoccatelli D, Clergue F (2016). Gender difference in career advancement and job satisfaction in Anaesthesia: a cross-sectional study. Eur J Anaesthesiol.

[41] Huang M, Ye L, Liang B, Ning C, Roth W, Jiang J (2016). Characterizing the HIV/AIDS epidemic in the United States and China. Int J Environ Res Public Health.

[42] Yuan FS, Liu L, Liu LH, Zeng YL, Zhang LL, He F (2021). Epidemiological and spatiotemporal analyses of HIV/AIDS prevalence among older adults in Sichuan, China between 2008 and 2019: a population-based study. Int J Infect Dis.

[43] Maslach C, Jackson SE (1986). MBI: maslach burnout inventory: manual research edition.

[44] Chaoping L, Kan S (2003). The influence of distributive justice and procedural justice on job burnout. Xin Li Xue Bao.

[45] Diener E, Wirtz D, Tov W, Kim-Prieto C, Choi D, Oishi S (2010). New well-being measures: short scales to assess flourishing and positive and negative feelings. Soc Indic Res.

[46] Wenjie D, Dan X (2016). Measuring adolescent flourishing: psychometric properties of flourishing scale in a sample of Chinese adolescents. J Psychoeduc Assess.

[47] Tong KK, Wang YY (2017). Validation of the flourishing scale and scale of positive and negative experience in a Chinese community sample. PLoS ONE.

[48] Hevey D (2018). Network analysis: a brief overview and tutorial. Health Psychol Behav Med.

[49] Fayers PM, Machin D (2008). Quality of life: the assessment, analysis and interpretation of patient-reported outcomes.

[50] Fava GA, Ruini C, Rafanelli C (2004). Psychometric theory is an obstacle to the progress of clinical research. Psychother Psychosom.

[51] Foygel R, Drton M (2010). Extended bayesian information criteria for gaussian graphical models.

[52] Ravikumar P, Wainwright MJ, Lafferty JD. High-dimensional Ising model selection using ℓ1-regularized logistic regression. Ann Stat. 2010;38(3):1287–319.

[53] Haslbeck J, Waldorp LJ (2018). How well do network models predict observations? On the importance of predictability in network models. Behav Res Methods.

[54] Epskamp S, Cramer AOJ, Waldorp LJ, Schmittmann VD, Borsboom D (2012). Qgraph: network visualizations of relationships in psychometric data. J Stat Softw.

[55] Epskamp S, Borsboom D, Fried EI (2018). Estimating psychological networks and their accuracy: a tutorial paper. Behav Res Methods.

[56] Jones PJ, Heeren A, McNally RJ (2017). Commentary: a network theory of mental disorders. Front Psychol.

[57] van Borkulo C, Boschloo L, Borsboom D, Penninx BWJH, Waldorp LJ, Schoevers RA (2015). Association of symptom network structure with the course of depression. JAMA Psychiat.

[58] Pe ML, Kircanski K, Thompson RJ, Bringmann LF, Tuerlinckx F, Mestdagh M (2015). Emotion-network density in major depressive disorder. Clin Psychol Sci.

[59] Boccaletti S, Latora V, Moreno Y, Chavez M, Hwang D (2006). Complex networks: structure and dynamics. Phys Rep.

[60] Cramer AO, Waldorp LJ, van der Maas HL, Borsboom D (2010). Comorbidity: a network perspective. Behav Brain Sci.

[61] Kim MH, Mazenga AC, Yu X, Simon K, Nyasulu P, Kazembe PN (2019). Factors associated with burnout amongst healthcare workers providing HIV care in Malawi. Plos One.

[62] Yang Y, Lu L, Chen T, Ye S, Kelifa MO, Cao N (2021). Healthcare worker's mental health and their associated predictors during the epidemic peak of covid-19. Psychol Res Behav Manag.

[63] Sovold LE, Naslund JA, Kousoulis AA, Saxena S, Qoronfleh MW, Grobler C (2021). Prioritizing the mental health and well-being of healthcare workers: an urgent global public health priority. Front Public Health.

[64] Oudiette D, Vinckier F, Bioud E, Pessiglione M (2019). A pavlovian account for paradoxical effects of motivation on controlling response vigour. Sci Rep.

[65] Redelinghuys K, Rothmann S, Botha E (2019). Flourishing-at-work: the role of positive organizational practices. Psychol Rep.

[66] Kelly-Hedrick M, Rodriguez MM, Ruble AE, Wright SM, Chisolm MS (2020). Measuring flourishing among internal medicine and psychiatry residents. J Grad Med Educ.

[67] Garcia-Iglesias JJ, Gomez-Salgado J, Fagundo-Rivera J, Romero-Martin M, Ortega-Moreno M, Navarro-Abal Y (2021). Predictive factors for burnout and work engagement levels among doctors and nurses: a systematic review. Rev Esp Salud Publica.

[68] Joanna S, Artur P (2012). Gender segregation of adolescent science career plans in 50 countries. Sci Educ.

[69] Slopen N, Williams DR, Fitzmaurice GM, Gilman SE (2011). Sex, stressful life events, and adult onset depression and alcohol dependence: are men and women equally vulnerable?. Soc Sci Med.

[70] Miers AC, Weeda WD, Blote AW, Cramer A, Borsboom D, Westenberg PM (2020). A cross-sectional and longitudinal network analysis approach to understanding connections among social anxiety components in youth. J Abnorm Psychol.

