# Supplementary material for: Occupational burnout, flourishing and job satisfaction among HIV/AIDS healthcare workers in Western China: a network analysis
Source: BMC Psychiatry. 2023 Aug 3;23:560. doi: 10.1186/s12888-023-04959-7 (PMC10398953; doi:10.1186/s12888-023-04959-7)
Supplement: Supplementary file 1 — Additional file 1: Table S1. Description of occupational burnout scale. Table S2. Description of flourishing scale. Table S3. Description of job satisfaction scale. Table S4. Description of the key variables used in this study. Table S5. Baseline characteristics and medians (IQR) for occupational burnout, job satisfaction and flourishing of the participants. Table S6. Weighted adjacency matrix. Figure S1. Flowchart of the study sample. Figure S2. Bootstrap 95% confidence intervals of the partial correlation coefficients. Figure S3. Estimation of edge weight differences by bootstrapped difference test. Figure S4. Bootstrap partial correlation coefficients difference test of the node’s strength. Figure S5. Stability of centrality indices by case dropping bootstrap in males and females. Figure S6. Bootstrap 95% confidence intervals of partial correlation coefficients of the network in males and females. Figure S7. Estimation of edge weight difference in males and females. Figure S8. Bootstrap partial correlation coefficients difference test of node’s strength in males and females. Figure S9. Comparison of network properties in males and females [file 12888_2023_4959_MOESM1_ESM.docx]

**Title** Occupational burnout, flourishing and job satisfaction among HIV/AIDS healthcare workers in western China: a network analysis

**Supplementary information**

**Table S1.** Description of occupational burnout scale 2

**Table S2.** Description of flourishing scale 3

**Table S3.** Description of job satisfaction scale 4

**Table S4.** Description of the key variables used in this study 5

**Table S5.** Baseline characteristics and medians (*IQR*) for occupational burnout, job satisfaction and flourishing of the participants 6

**Table S6.** Weighted adjacency matrix 7-8

**Figure S1**. Flowchart of the study sample 1

**Figure S2.** Bootstrap 95% confidence intervals of the partial correlation coefficients 9-10

**Figure S3.** Estimation of edge weight differences by bootstrapped difference test 11

**Figure S4.** Bootstrap partial correlation coefficients difference test of the node’s strength 12

**Figure S5.** Stability of centrality indices by case dropping bootstrap in males and females 13

**Figure S6.** Bootstrap 95% confidence intervals of partial correlation coefficients of the network in males and females 14-15

**Figure S7**. Estimation of edge weight difference in males and females 16-17

**Figure S8.** Bootstrap partial correlation coefficients difference test of node’s strength in males and females 18-19

**Figure S9.** Comparison of network properties in males and females 20

Designated Hospital for treatment (n=105)

County Governments (n=95)

Community Health Service Centers (n=591)

Others ^1^ (n=25)

Centers for Disease Control and Prevention (n=91)

907 participants included in the analysis

Excluded for the following reasons (n=15)

12 participants with missing data on relevant variables used in the analysis

3 participants with a contradiction between age and job tenure

922 individuals recruited enrolled participants

**Figure S1. Flowchart of the study sample**

^1^ Others are self-selected designation that indicates the institution is not listed.

**Table S1. Description of occupational burnout scale**

| **Item** | **mean ± SD** |
| --- | --- |
| I feel my emotions have been exhausted at work | 3.14±1.05 |
| I feel that I am exhausted | 3.02±1.01 |
| I feel like that I have done my best | 3.20±1.09 |
| I become indifferent to people after taking this job | 2.27±0.99 |
| I am worried that the job will make me emotionally numb | 2.36±1.01 |
| I treat some people without emotion in workplace | 2.17±0.92 |
| I don't care what happens to other people in workplace | 2.47±1.03 |
| Doing a work of dealing with people all day is a test of me | 3.28±1.10 |
| Dealing with people directly adds a lot of pressure to me | 2.77±1.03 |
| I often get frustrated at work | 2.75±0.98 |
| I feel that people in workplace will blame me for some of their problems | 2.63±1.01 |

SD: standard deviation

**Table S2. Description of flourishing scale**

| **Item** | **mean ± SD** |
| --- | --- |
| I lead a purposeful and meaningful life | 5.07±1.15 |
| My social relationships are supportive and rewarding | 4.82±1.30 |
| I am interested in my daily activities | 4.91±1.24 |
| I actively contribute to the happiness and well-being of others | 5.53±1.01 |
| I am competent and capable in the activities that are important to me | 5.66±1.11 |
| I am a good person and live a good life | 5.28±1.39 |
| I am optimistic about my future | 5.27±1.41 |
| People respect me | 5.43±1.33 |

SD: standard deviation

**Table S3. Description of job satisfaction scale**

| **Dimensions** | **Item** | **mean ± SD** |
| --- | --- | --- |
| Satisfaction with job itself | I like the current work | 4.07±1.12 |
|  | My interests match well with my major | 3.61±1.34 |
|  | I think I am suitable for the current position | 4.00±1.16 |
|  | I am willing to receive training to improve the skills required for my job | 4.60±0.78 |
|  | I am clear about my job responsibilities and rights | 4.58±0.73 |
|  | I feel a sense of accomplishment in my work | 4.10±1.05 |
|  | I think I am qualified for my present job | 4.29±0.95 |
|  | I don't think I take much risk in this profession | 2.73±1.46 |
|  | I don't think there is much pressure at work | 2.52±1.42 |
|  | I don't think I have much challenge in this profession | 2.33±1.32 |
| Job environment satisfaction | I think the facilities of this unit can meet the needs of the work | 3.58±1.35 |
|  | I have good relationships with members of the department | 4.64±0.69 |
|  | I have good communications with other department members | 4.53±0.74 |
|  | I have good working relationships and communications with my superiors | 4.47±0.78 |
|  | I think my department have a good spirit of cooperation with each other | 4.54±0.76 |
|  | I think the working environment in the department is very good | 4.13±1.09 |
| Job reward satisfaction | The work I do is well received by my peers | 4.22±0.91 |
|  | I think we have more training opportunities than our peers | 3.55±1.23 |
|  | The existing training is indeed helpful for my practical working ability | 4.41±0.85 |
|  | I think I have a fair spirit and development opportunities in the unit | 3.79±1.22 |
|  | I think there are opportunities for promotion in this unit | 3.34±1.29 |
|  | I am quite satisfied with my current income | 2.60±1.38 |
|  | I am satisfied with the welfare and subsidies of the unit | 2.80±1.40 |
|  | I think my payment is less than the return | 3.23±1.45 |

SD: standard deviation

**Table S4. Description of the key variables used in this study**

| **Covariates** | **Categories** | **Description** |
| --- | --- | --- |
| ***Socio-demographic characteristics*** |  |  |
| Age (years) | 18-29 years, 30-39 years, 40-49 years, ≥ 50 years | - |
| Sex | Female, male | - |
| Marital status | Unmarried, married/living together, divorced/widowed/living separately | - |
| Educational level | High school or below, junior college, undergraduate or above | - |
| Personal monthly income (RMB） | <3000 RMB, 3000-4000 RMB, 4000-5000 RMB, ≥5000 RMB | - |
| Living situation | Alone, with friends or others, with parents | - |
| ***Work-related characteristics*** |  |  |
| Type of institution | Centers for Disease Control and Prevention, Designated Hospital for treatment, County Governments, Community Health Service Centers, others | Others are self-selected designation that indicates the institution is not listed. |

**Table S5. Baseline characteristics and medians (*IQR*) for occupational burnout, job satisfaction and flourishing of the participants**

| **Variables** | **N** | **Occupational burnout**  **(Median [*IQR*])** | **Job satisfaction**  **(Median [*IQR*])** | **Flourishing**  **(Median [*IQR*])** |
| --- | --- | --- | --- | --- |
| **Age (years)** |  |  |  |  |
| 18-29 | 224 | 31.0 (25.0, 34.0) | 89.0 (78.0, 99.0) | 40.0 (32.0, 48.0) |
| 30-39 | 300 | 29.0 (24.0, 34.0) | 94.0 (82.0, 103.0) | 44.0 (36.0, 48.0) |
| 40-49 | 253 | 30.0 (26.0, 34.0) | 92.0 (82.0, 102.0) | 44.0 (38.0, 48.0) |
| ≥50 | 130 | 31.0 (27.0, 34.0) | 92.0 (78.0, 101.8) | 44.0 (39.3, 48.0) |
| **Sex** |  |  |  |  |
| Female | 627 | 30.0 (25.0, 33.0) | 92.0 (80.5, 101.0) | 43.0 (35.0, 48.0) |
| Male | 280 | 31.0 (27.0, 35.0) | 93.0 (80.8, 103.0) | 44.0 (37.0, 48.0) |
| **Marital status** |  |  |  |  |
| Unmarried | 118 | 31.0 (23.3, 35.0) | 88.0 (76.3, 102.0) | 40.0 (32.0, 48.0) |
| Married/Living together | 706 | 30.0 (25.0, 34.0) | 92.0 (81.0, 102.0) | 44.0 (37.0, 48.0) |
| Divorce/Widowed/Living separately | 83 | 30.0 (26.0, 33.0) | 90.0 (78.0, 97.5) | 42.0 (34.5, 47.5) |
| **Educational level** |  |  |  |  |
| High school or below | 123 | 30.0 (26.0, 33.5) | 92.0 (82.5, 101.5) | 44.0 (36.0, 48.0) |
| Junior college | 449 | 31.0 (26.0, 35.0) | 91.0 (79.0, 102.0) | 43.0 (35.0, 48.0) |
| Undergraduate or above | 335 | 29.0 (24.0, 33.0) | 92.0 (81.0, 102.0) | 43.0 (36.0, 48.0) |
| **Personal monthly income (RMB）** |  |  |  |  |
| <3000 | 253 | 30.0 (25.0, 33.0) | 90.0 (79.0, 101.0) | 42.0 (35.0, 48.0) |
| 3000-4000 | 304 | 30.0 (24.0, 34.0) | 90.0 (79.0, 102.0) | 43.0 (34.0, 48.0) |
| 4000-5000 | 176 | 31.0 (27.0, 34.0) | 92.0 (81.0, 99.3) | 43.0 (37.0, 48.0) |
| ≥5000 | 174 | 29.0 (25.0, 34.0) | 96.0 (85.0, 104.0) | 45.0 (39.0, 48.0) |
| **Living situation** |  |  |  |  |
| Alone | 74 | 31.0 (27.0, 37.0) | 88.5 (75.0, 96.8) | 41.5 (32.0, 46.8) |
| With friends or others | 41 | 30.0 (27.0, 35.0) | 82.0 (70.0, 98.0) | 41.0 (32.0, 48.0) |
| With parents | 792 | 30.0 (25.0, 34.0) | 92.0 (82.0, 102.0) | 44.0 (36.0, 48.0) |
| **Types of institution** |  |  |  |  |
| CDC | 91 | 31.0 (25.5, 34.0) | 85.0 (75.5, 96.0) | 40.0 (34.0, 47.0) |
| Designated Hospital for treatment | 105 | 28.0 (23.0, 33.0) | 96.0 (89.0, 105.0) | 45.0 (36.0, 49.0) |
| County Governments | 95 | 28.0 (22.5, 33.0) | 96.0 (81.0, 108.0) | 46.0 (37.5, 48.0) |
| Community Health Service Centers | 591 | 31.0 (26.0, 35.0) | 92.0 (80.0, 102.0) | 43.0 (35.0, 48.0) |
| Others ^1^ | 25 | 30.0 (25.0, 33.0) | 93.0 (87.0, 108.0) | 45.0 (40.0, 48.0) |

Abbreviations: *IQR*, interquartile range; CDC, Centers for Disease Control and Prevention. RMB: renminbi

^1^ Others is a self-selected designation that indicates the institution is not listed.

**Table S6. Weighted adjacency matrix**

|  | W1 | W2 | W3 | H1 | H2 | H3 | H4 | H5 | H6 | H7 | H8 | E1 | E2 | E3 | E4 | E5 | E6 | E7 | E8 | E9 | E10 | E11 |
| --- | --- | --- | --- | --- | --- | --- | --- | --- | --- | --- | --- | --- | --- | --- | --- | --- | --- | --- | --- | --- | --- | --- |
| W1 | 0 | 0.20 | 0.38 | 0 | 0 | 0.11 | 0.01 | 0 | 0 | 0 | 0.04 | 0 | -0.05 | 0 | 0.04 | 0 | 0 | 0.01 | 0 | 0 | 0 | 0 |
| W2 | 0.20 | 0 | 0.32 | 0 | 0 | 0.03 | 0 | 0 | 0 | 0.04 | 0.01 | -0.02 | -0.02 | 0 | 0 | -0.03 | 0 | 0 | 0.06 | 0 | -0.03 | 0 |
| W3 | 0.38 | 0.32 | 0 | 0.01 | 0.07 | 0.10 | 0 | -0.04 | 0.05 | 0 | 0.03 | 0 | 0 | 0 | 0.03 | 0 | 0.02 | 0 | 0 | 0 | -0.04 | 0 |
| H1 | 0 | 0 | 0.01 | 0 | 0.29 | 0.30 | 0.08 | 0 | 0.07 | 0.20 | 0.02 | -0.03 | 0 | -0.02 | 0.03 | 0 | 0 | 0 | 0.01 | 0.02 | -0.05 | 0 |
| H2 | 0 | 0 | 0.07 | 0.29 | 0 | 0.24 | 0.11 | 0 | 0 | 0 | 0.02 | 0 | 0.01 | 0 | 0 | 0 | 0.02 | 0 | 0 | 0 | 0 | -0.01 |
| H3 | 0.11 | 0.03 | 0.10 | 0.30 | 0.24 | 0 | 0.22 | -0.02 | 0.01 | 0.10 | 0.09 | 0 | -0.05 | 0 | 0 | 0 | 0 | 0.03 | 0 | 0 | 0 | 0 |
| H4 | 0.01 | 0 | 0 | 0.08 | 0.11 | 0.22 | 0 | 0.39 | 0.03 | 0 | 0.01 | 0 | 0 | 0.07 | -0.08 | -0.02 | -0.05 | 0 | 0 | 0.04 | 0.04 | 0 |
| H5 | 0 | 0 | -0.04 | 0 | 0 | -0.02 | 0.39 | 0 | 0.25 | 0.01 | 0.22 | 0.06 | 0.02 | 0 | 0 | 0 | 0 | -0.07 | 0.05 | -0.06 | 0 | 0 |
| H6 | 0 | 0 | 0.05 | 0.07 | 0 | 0.01 | 0.03 | 0.25 | 0 | 0.49 | 0.15 | 0 | 0 | 0 | 0 | 0 | 0 | 0 | 0.02 | 0 | 0 | 0 |
| H7 | 0 | 0.04 | 0 | 0.20 | 0 | 0.10 | 0 | 0.01 | 0.49 | 0 | 0.31 | -0.01 | 0 | 0 | 0 | 0 | 0 | 0 | 0 | 0 | -0.02 | 0 |
| H8 | 0.04 | 0.01 | 0.03 | 0.02 | 0.02 | 0.09 | 0.01 | 0.22 | 0.15 | 0.31 | 0 | 0 | 0 | 0 | 0 | 0 | -0.07 | 0.05 | 0.02 | 0 | -0.03 | 0 |
| E1 | 0 | -0.02 | 0 | -0.03 | 0 | 0 | 0 | 0.06 | 0 | -0.01 | 0 | 0 | 0.55 | 0.19 | 0.02 | 0 | 0.02 | 0 | 0.07 | 0 | 0.10 | 0.01 |
| E2 | -0.05 | -0.02 | 0 | 0 | 0.01 | -0.05 | 0 | 0.02 | 0 | 0 | 0 | 0.55 | 0 | 0.45 | 0.12 | 0.02 | -0.06 | -0.01 | 0 | 0.04 | 0.04 | 0 |
| E3 | 0 | 0 | 0 | -0.02 | 0 | 0 | 0.07 | 0 | 0 | 0 | 0 | 0.19 | 0.45 | 0 | 0 | 0.01 | 0 | 0.05 | 0.05 | 0.01 | 0 | 0.07 |
| E4 | 0.04 | 0 | 0.03 | 0.03 | 0 | 0 | -0.08 | 0 | 0 | 0 | 0 | 0.02 | 0.12 | 0 | 0 | 0.59 | 0.13 | 0 | -0.14 | 0.06 | 0 | 0.02 |
| E5 | 0 | -0.03 | 0 | 0 | 0 | 0 | -0.02 | 0 | 0 | 0 | 0 | 0 | 0.02 | 0.01 | 0.59 | 0 | 0.27 | 0 | 0 | 0.09 | 0.09 | 0 |
| E6 | 0 | 0 | 0.02 | 0 | 0.02 | 0 | -0.05 | 0 | 0 | 0 | -0.07 | 0.02 | -0.06 | 0 | 0.13 | 0.27 | 0 | 0.38 | 0 | 0.02 | 0.01 | 0.17 |
| E7 | 0.01 | 0 | 0 | 0 | 0 | 0.03 | 0 | -0.07 | 0 | 0 | 0.05 | 0 | -0.01 | 0.05 | 0 | 0 | 0.38 | 0 | 0.16 | 0.03 | 0.07 | 0 |
| E8 | 0 | 0.06 | 0 | 0.01 | 0 | 0 | 0 | 0.05 | 0.02 | 0 | 0.02 | 0.07 | 0 | 0.05 | -0.14 | 0 | 0 | 0.16 | 0 | 0.35 | 0.05 | 0.02 |
| E9 | 0 | 0 | 0 | 0.02 | 0 | 0 | 0.04 | -0.06 | 0 | 0 | 0 | 0 | 0.04 | 0.01 | 0.06 | 0.09 | 0.02 | 0.03 | 0.35 | 0 | 0.37 | 0.12 |
| E10 | 0 | -0.03 | -0.04 | -0.05 | 0 | 0 | 0.04 | 0 | 0 | -0.02 | -0.03 | 0.10 | 0.04 | 0 | 0 | 0.09 | 0.01 | 0.07 | 0.05 | 0.37 | 0 | 0.33 |
| E11 | 0 | 0 | 0 | 0 | -0.01 | 0 | 0 | 0 | 0 | 0 | 0 | 0.01 | 0 | 0.07 | 0.02 | 0 | 0.17 | 0 | 0.02 | 0.12 | 0.33 | 0 |

Weighted adjacency matrix based on three job satisfaction dimensions (10 direct job satisfaction items, 6 job environment satisfaction items and 8 job reward satisfaction items), eight flourishing items, and eleven occupational burnout items to represent the partial correlation coefficients between nodes in the network.

E1: feeling emotional exhaustion at work; E2: feeling exhausted at work; E3: feeling can do everything; E4: become cold after taking this job; E5: be afraid job will make emotionally numbness; E6: having no emotion for some patients; E7: don't care what happens to some patients; E8: finding working with people all day is a test; E9: feeling a lot of pressure when dealing with people; E10: feeling frustrated at work; E11: feeling patients would blame for some of their problems. H1: leading a purposeful and meaningful life; H2: having supportive and rewarding social relationships ; H3: interested in daily activities; H4: contributing to the happiness and well-being of others; H5: be competent and capable in the activities; H6: be a good person and live a good life; H7: be optimistic about future; H8: feeling respectable. W: three dimensions of job satisfaction, including W1, satisfaction with job itself; W2, job environment satisfaction; W3, job reward satisfaction.


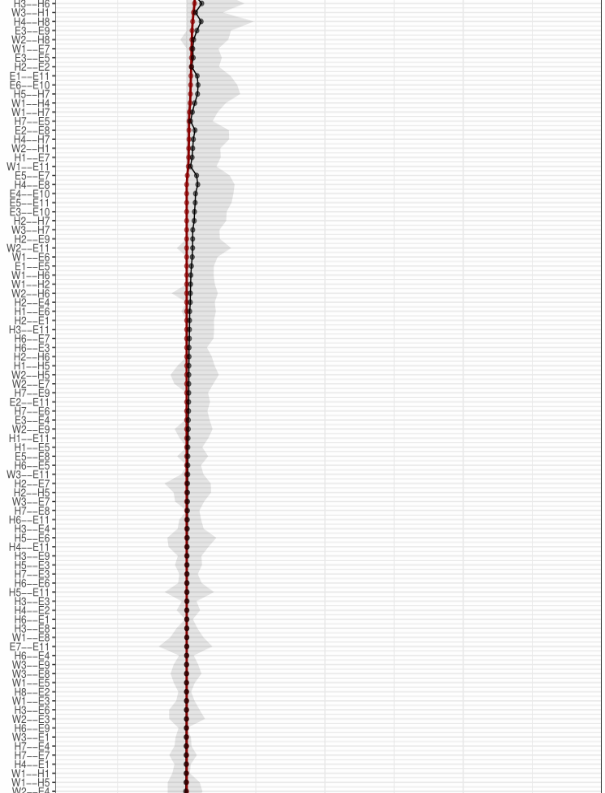

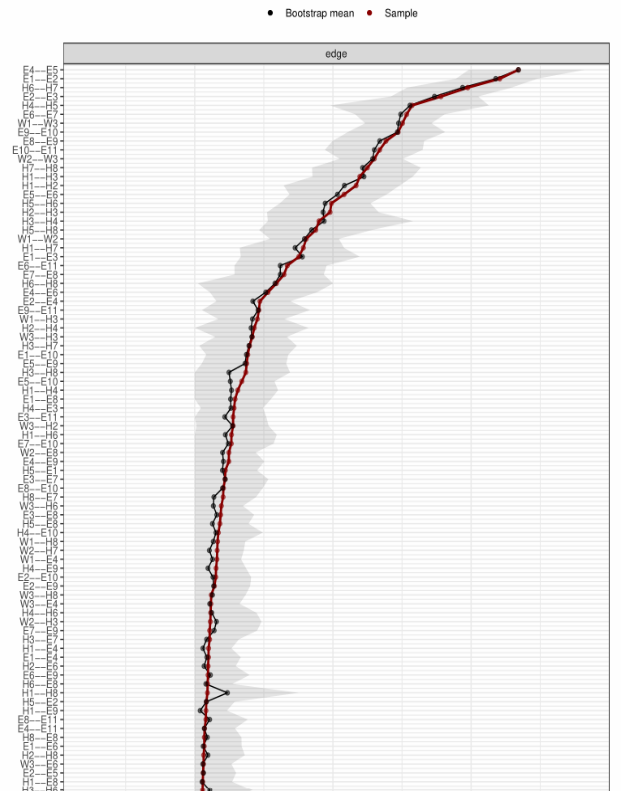

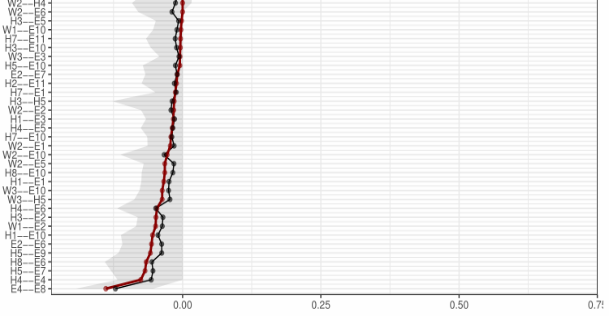


**Figure S2.** Bootstrap 95% confidence intervals of the partial correlation coefficients of the network shown in Figure 1 (The bootstrap was repeated 1000 times). Individual edges are represented by a horizontal grey line, the original edges’ weight and bootstrap edges’ weight are indicated by the red line and black line, respectively. The grey area represents the bootstrap 95% confidence interval.

E1: feeling emotional exhaustion at work; E2: feeling exhausted at work; E3: feeling can do everything; E4: become cold after taking this job; E5: be afraid job will make emotionally numbness; E6: having no emotion for some patients; E7: don't care what happens to some patients; E8: finding working with people all day is a test; E9: feeling a lot of pressure when dealing with people; E10: feeling frustrated at work; E11: feeling patients would blame for some of their problems. H1: leading a purposeful and meaningful life; H2: having supportive and rewarding social relationships; H3: interested in daily activities; H4: contributing to the happiness and well-being of others; H5: be competent and capable in the activities; H6: be a good person and live a good life; H7: be optimistic about future; H8: feeling respectable. W: three dimensions of job satisfaction, including W1, satisfaction with job itself; W2, job environment satisfaction; W3, job reward satisfaction.


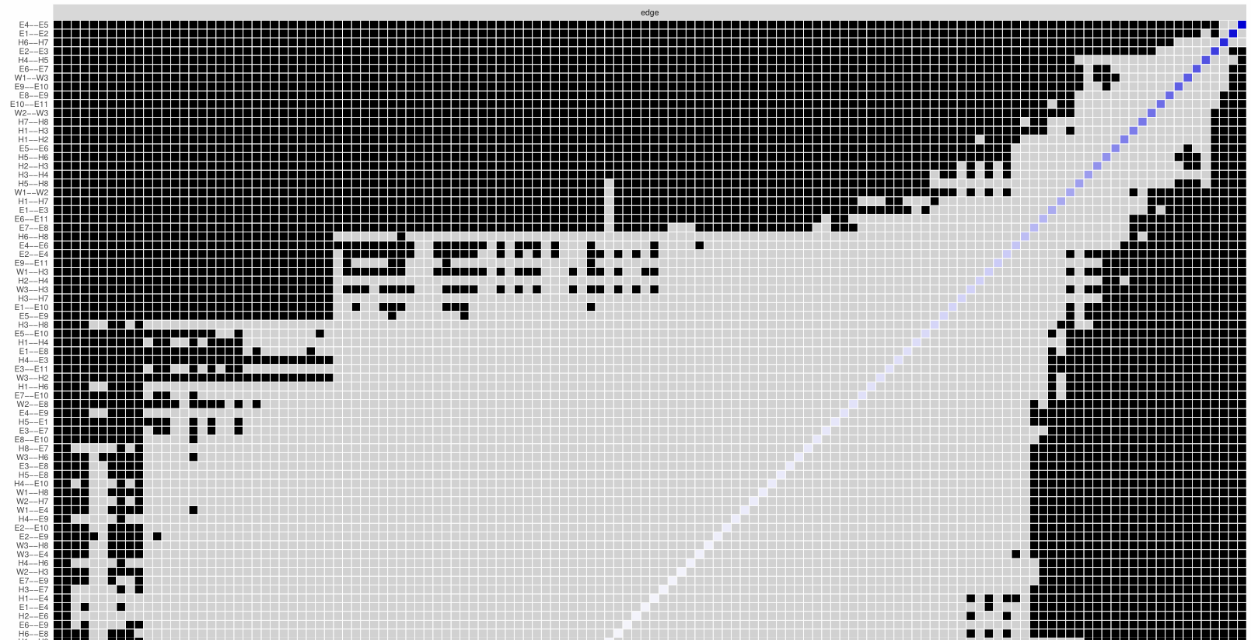

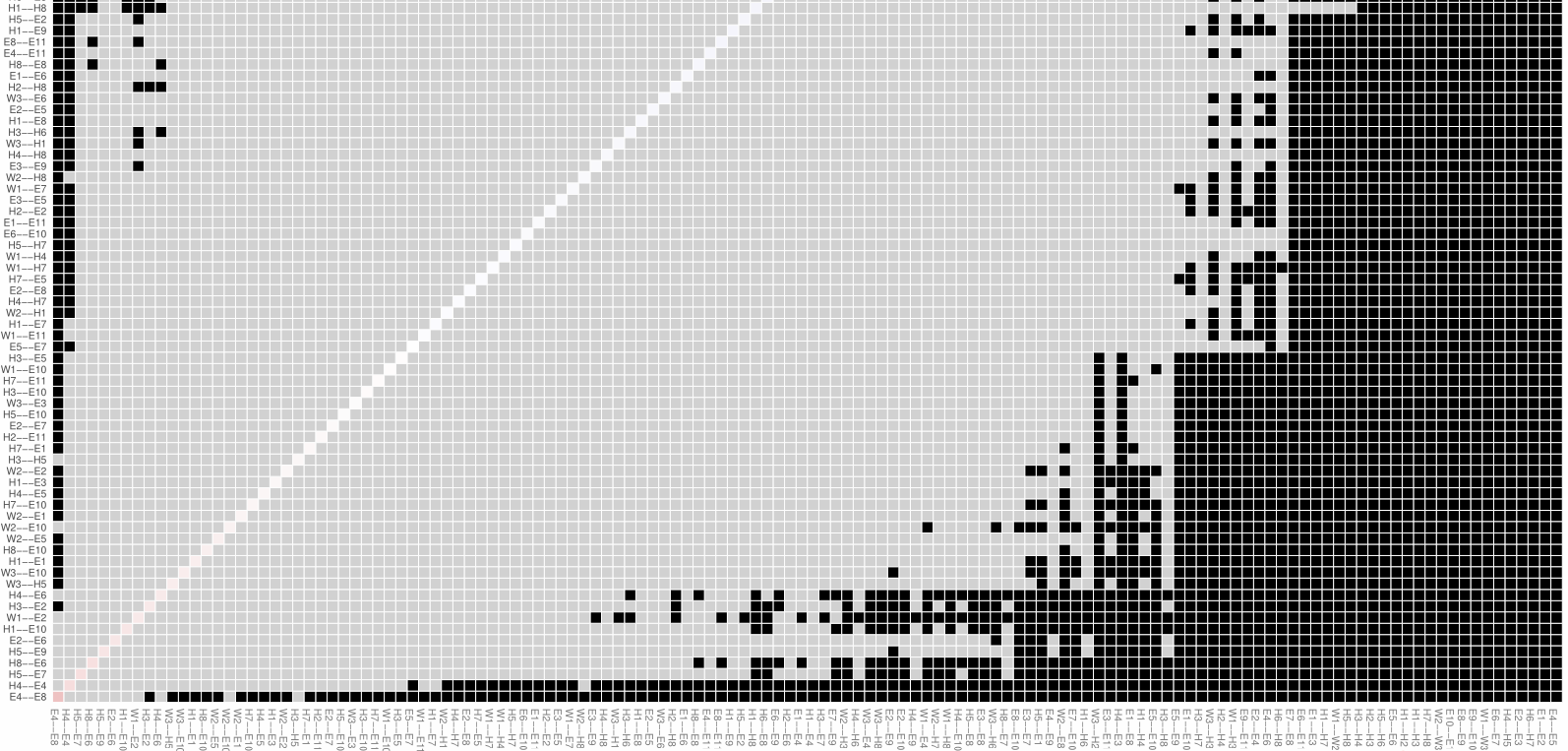


**Figure S3.** Estimation of partial correlation coefficients difference by bootstrapped difference test (The bootstrap was repeated 1000 times). Bootstrapped difference tests between partial correlation coefficients in the network. Gray boxes indicate edges that do not differ from one-another significantly. Black boxes represent edges with significant difference from one another (*α* = 0.05). Blue boxes in the edge-weight plot correspond to positive correlations and red boxes to negative correlations.

E1: feeling emotional exhaustion at work; E2: feeling exhausted at work; E3: feeling can do everything; E4: become cold after taking this job; E5: be afraid job will make emotionally numbness; E6: having no emotion for some patients; E7: don't care what happens to some patients; E8: finding working with people all day is a test; E9: feeling a lot of pressure when dealing with people; E10: feeling frustrated at work; E11: feeling patients would blame for some of their problems. H1: leading a purposeful and meaningful life; H2: having supportive and rewarding social relationships ; H3: interested in daily activities; H4: contributing to the happiness and well-being of others; H5: be competent and capable in the activities; H6: be a good person and live a good life; H7: be optimistic about future; H8: feeling respectable. W: three dimensions of job satisfaction, including W1, satisfaction with job itself; W2, job environment satisfaction; W3, job reward satisfaction.


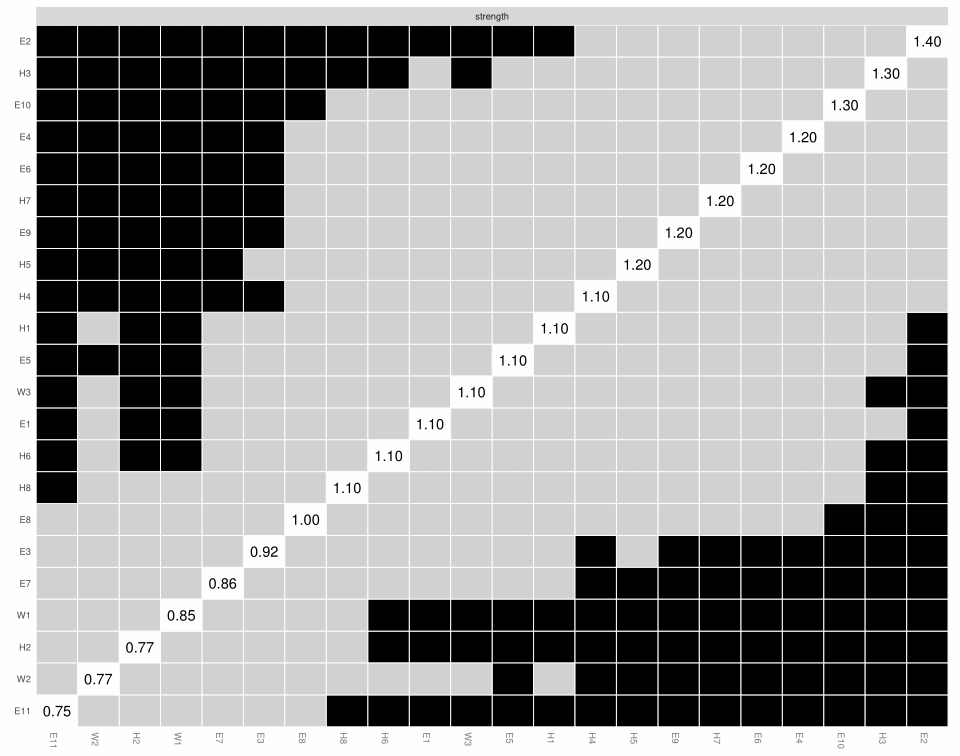


**Figure S4**. Bootstrap partial correlation coefficients difference test of the node’s strength (The bootstrap was repeated 1000 times). Grey boxes indicate non-significant differences while black boxes represent significant differences in strength between two variables (*α* = 0.05). The numerical magnitude of each nodes’ strength is indicated in the diagonal boxes.

E1: feeling emotional exhaustion at work; E2: feeling exhausted at work; E3: feeling can do everything; E4: become cold after taking this job; E5: be afraid job will make emotionally numbness; E6: having no emotion for some patients; E7: don't care what happens to some patients; E8: finding working with people all day is a test; E9: feeling a lot of pressure when dealing with people; E10: feeling frustrated at work; E11: feeling patients would blame for some of their problems. H1: leading a purposeful and meaningful life; H2: having supportive and rewarding social relationships ; H3: interested in daily activities; H4: contributing to the happiness and well-being of others; H5: be competent and capable in the activities; H6: be a good person and live a good life; H7: be optimistic about future; H8: feeling respectable. W: three dimensions of job satisfaction, including W1, satisfaction with job itself; W2, job environment satisfaction; W3, job reward satisfaction.


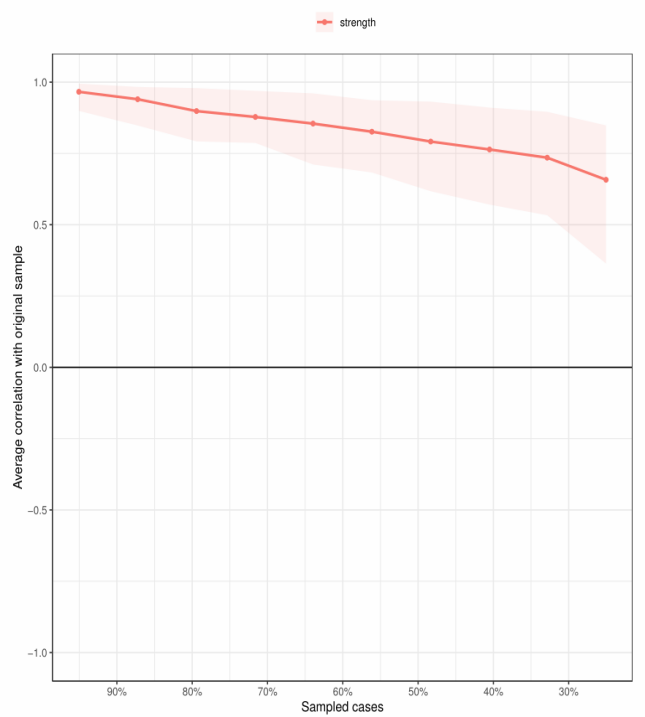

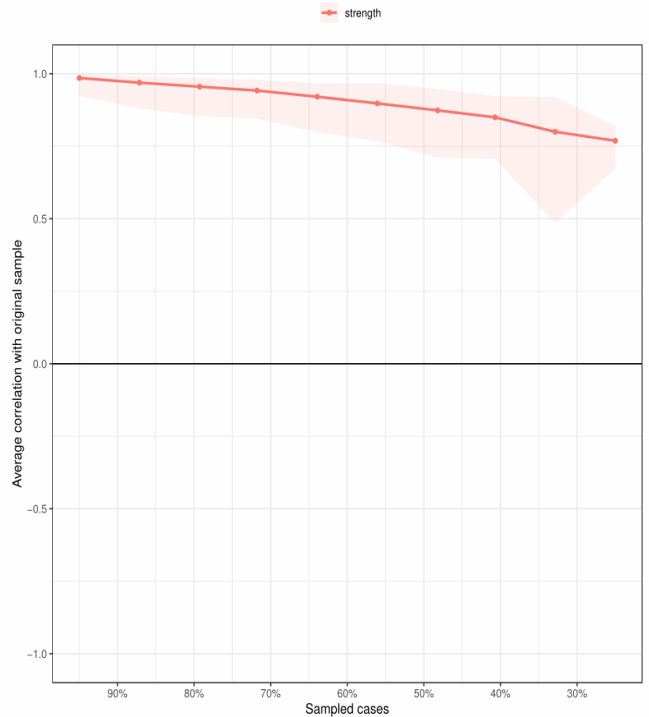


**(A)**

**(B)**

**Figure S5**. Stability of centrality indices by case dropping bootstrap in males **(A)** and females **(B)** (The bootstrap was repeated 1000 times). The x-axis represents the percentage of cases of original sample used at each step. The y-axis represents the average of correlations between the centrality indices from the original network and the centrality indices from the networks that were re-estimated after dropping increasing percentages of cases. Each line indicates the correlations of strength, while areas indicate 95% confidence intervals.


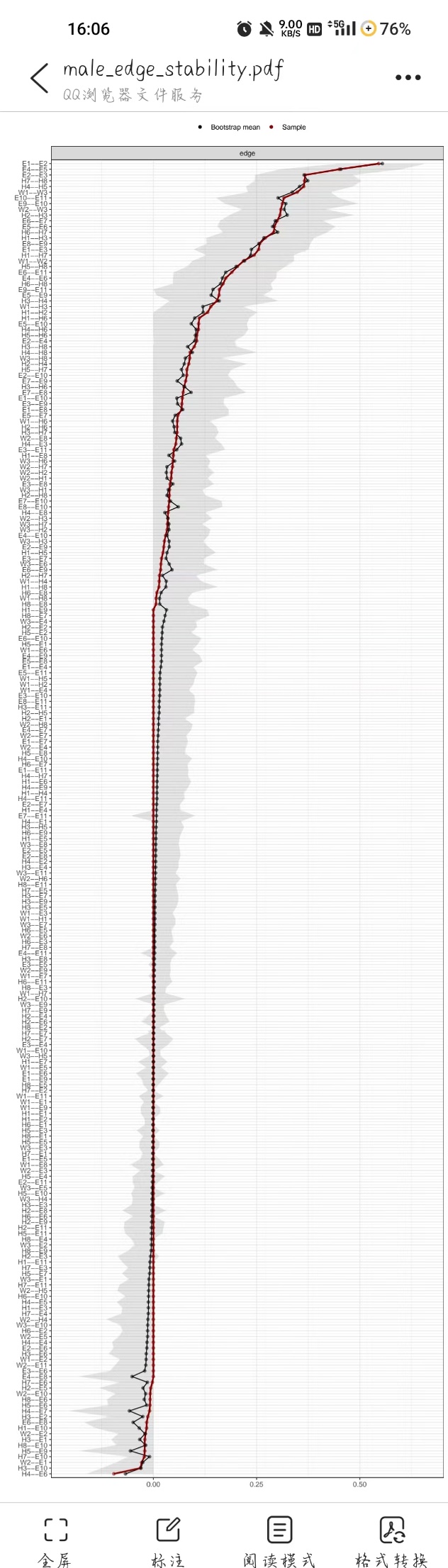


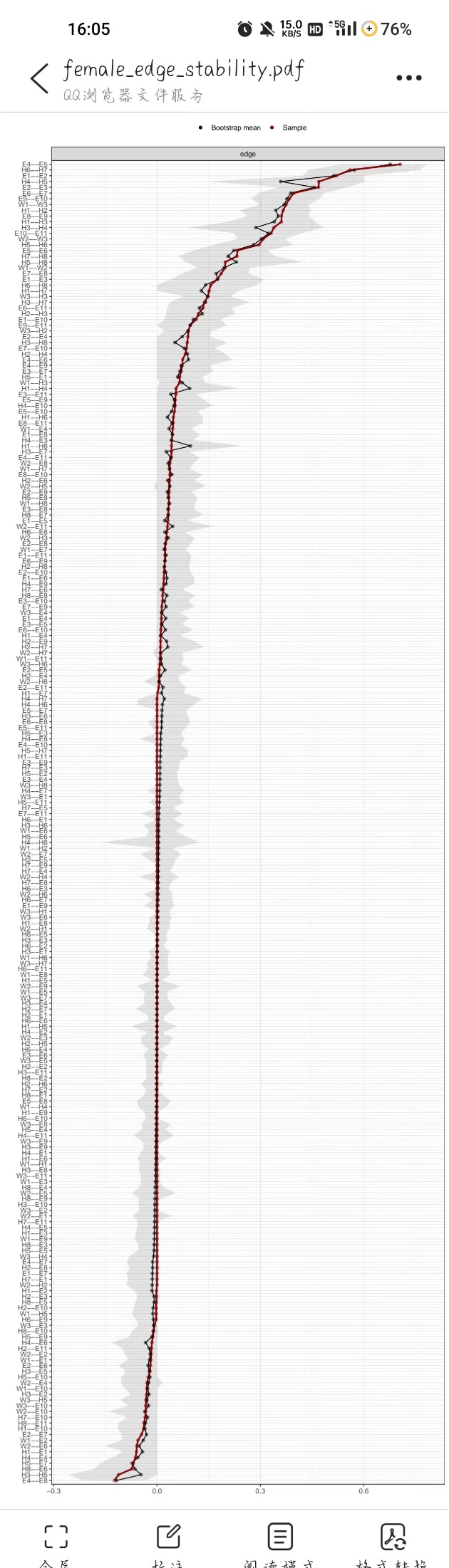


**(A)**

**(B)**

**Figure S6.** Bootstrap 95% confidence intervals of the partial correlation coefficients of the network in males **(A)** and femalse **(B)** (The bootstrap was repeated 1000 times). Individual edges are represented by a horizontal grey line, the original edges’ weight and bootstrap edges’ weight are indicated by the red line and black line, respectively. The grey area represents the bootstrap 95% confidence intervals.

E1: feeling emotional exhaustion at work; E2: feeling exhausted at work; E3: feeling can do everything; E4: become cold after taking this job; E5: be afraid job will make emotionally numbness; E6: having no emotion for some patients; E7: don't care what happens to some patients; E8: finding working with people all day is a test; E9: feeling a lot of pressure when dealing with people; E10: feeling frustrated at work; E11: feeling patients would blame for some of their problems. H1: leading a purposeful and meaningful life; H2: having supportive and rewarding social relationships ; H3: interested in daily activities; H4: contributing to the happiness and well-being of others; H5: be competent and capable in the activities; H6: be a good person and live a good life; H7: be optimistic about future; H8: feeling respectable. W: three dimensions of job satisfaction, including W1, satisfaction with job itself; W2, job environment satisfaction; W3, job reward satisfaction.


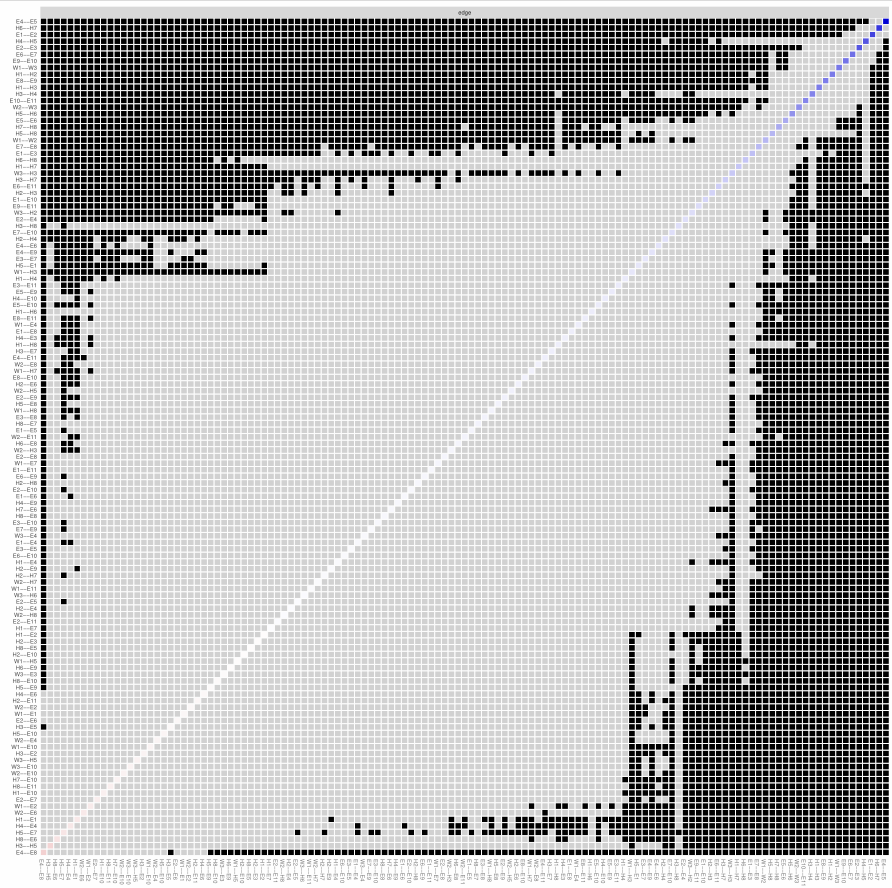


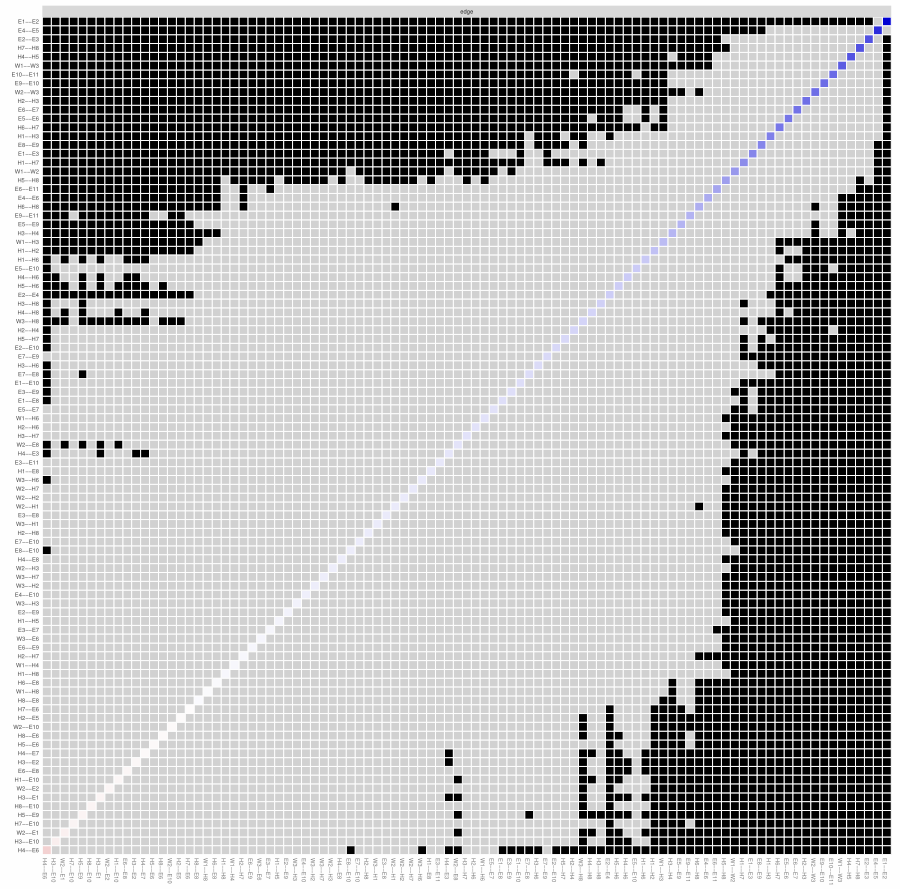


**(A)**

**(B)**

**Figure S7**. Estimation of partial correlation coefficients difference by bootstrapped difference test in males **(A)** and females **(B)** (The bootstrap was repeated 1000 times). Bootstrapped difference tests between partial correlation coefficients in the network. Gray boxes indicate edges that do not differ from one-another significantly, while black boxes represent edges with significant difference from one another (*α* = 0.05). Blue boxes in the edge-weight plot correspond to positive correlations and red boxes to negative correlations.

E1: feeling emotional exhaustion at work; E2: feeling exhausted at work; E3: feeling can do everything; E4: become cold after taking this job; E5: be afraid job will make emotionally numbness; E6: having no emotion for some patients; E7: don't care what happens to some patients; E8: finding working with people all day is a test; E9: feeling a lot of pressure when dealing with people; E10: feeling frustrated at work; E11: feeling patients would blame for some of their problems. H1: leading a purposeful and meaningful life; H2: having supportive and rewarding social relationships ; H3: interested in daily activities; H4: contributing to the happiness and well-being of others; H5: be competent and capable in the activities; H6: be a good person and live a good life; H7: be optimistic about future; H8: feeling respectable. W: three dimensions of job satisfaction, including W1, satisfaction with job itself; W2, job environment satisfaction; W3, job reward satisfaction.

**(A)**

**(B)**


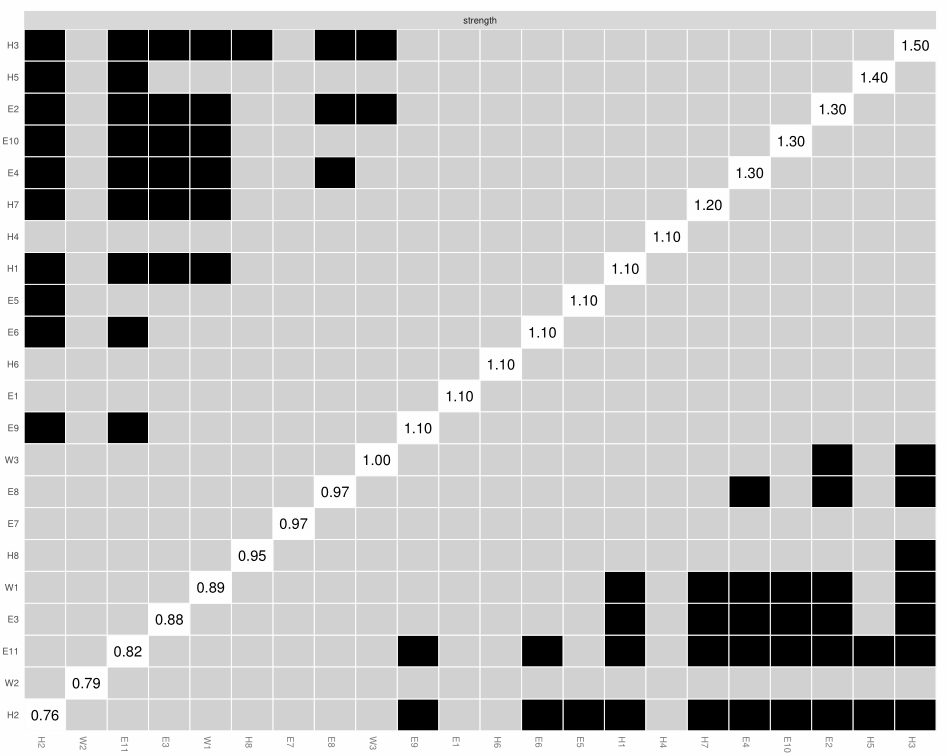

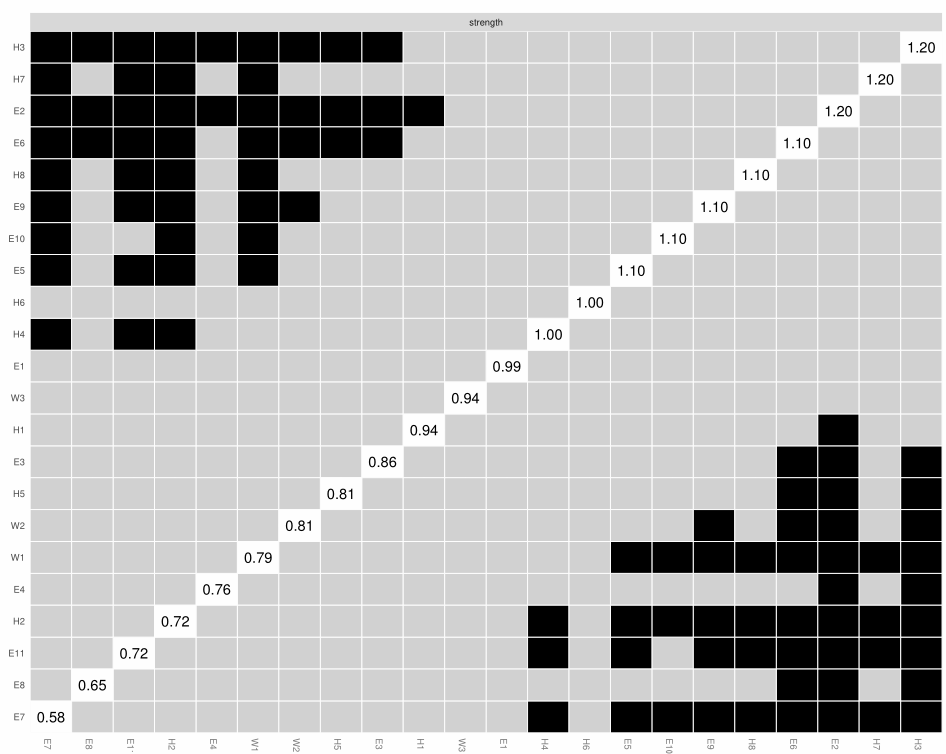


**Figure S8**. Bootstrap partial correlation coefficients difference test of the node’s strength in males **(A)** and females **(B)** (The bootstrap was repeated 1000 times). Grey boxes indicate non-significant differences， while black boxes represent significant differences in strength between two variables (*α* = 0.05). The numerical magnitude of each nodes’ strength is indicated in the diagonal boxes.

E1: feeling emotional exhaustion at work; E2: feeling exhausted at work; E3: feeling can do everything; E4: become cold after taking this job; E5: be afraid job will make emotionally numbness; E6: having no emotion for some patients; E7: don't care what happens to some patients; E8: finding working with people all day is a test; E9: feeling a lot of pressure when dealing with people; E10: feeling frustrated at work; E11: feeling patients would blame for some of their problems. H1: leading a purposeful and meaningful life; H2: having supportive and rewarding social relationships ; H3: interested in daily activities; H4: contributing to the happiness and well-being of others; H5: be competent and capable in the activities; H6: be a good person and live a good life; H7: be optimistic about future; H8: feeling respectable. W: three dimensions of job satisfaction, including W1, satisfaction with job itself; W2, job environment satisfaction; W3, job reward satisfaction.


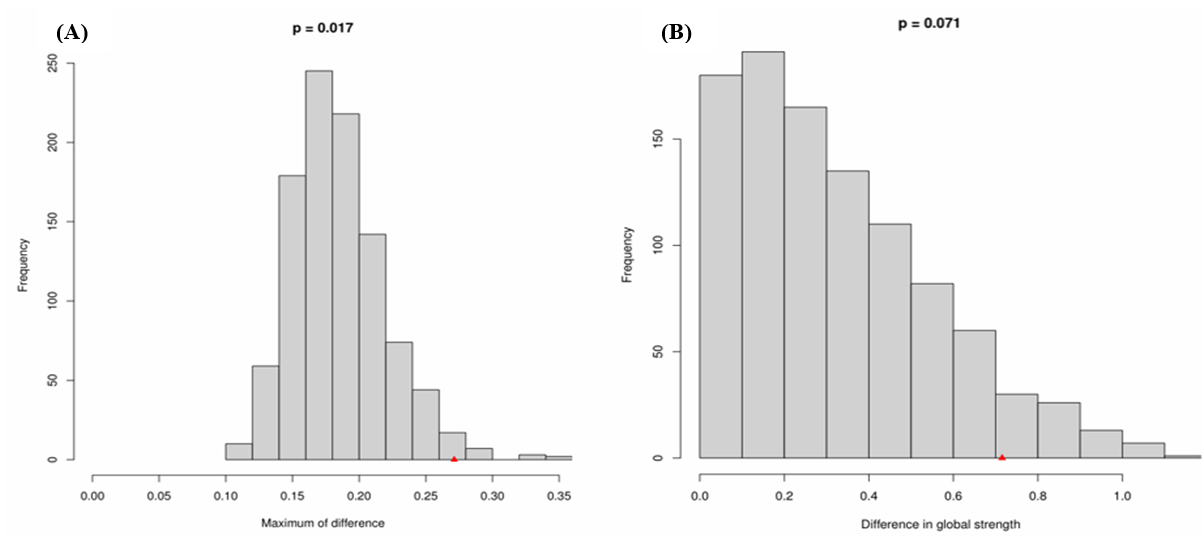
**Figure S9.** Comparison of network properties in males and females. **(A)** Plot of bootstrap value of the maximum of difference in distribution of partial correlation coefficients (1000 permutations). The difference was significant (*M*=0.271, *P*=0.017). **(B)** Plot of bootstrap value of the difference in network global strength. The difference was not significant (network strength among females: 10.62984; network strength among males: 9.914365; S: 0.7155, *P*=0.071). The red triangle indicates the test statistic based on the observed (real) data. Invariance in edges weights was tested using the permutation test, generating sets of p values for each edge-edge comparison. Holm-Bonferroni corrected *P* values were all >0.05 indicating absence of significant differences.
